# Testing the Langer–Bar-on–Miller–Akcasu Equation for the Time Evolution of the Structure Factor during Polymeric Spinodal Decomposition and Dissolution

**DOI:** 10.1021/acs.macromol.6c00098

**Published:** 2026-04-20

**Authors:** Matthew Jones, Nigel Clarke

**Affiliations:** † School of Mathematical and Physical Sciences, University of Sheffield, Hicks Building, Hounsfield Road, Sheffield, S3 7RH, United Kingdom; ‡ 7315 Now at Infinitesima Limited, Oxford, OX14 1RG, United Kingdom

## Abstract

Thermally induced spinodal decomposition and dissolution offer a route between miscible and immiscible microstructures in polymer blends, affecting the properties of the resulting material. Information about the microstructure can be deduced from measurements of the structure factor obtained using small-angle scattering. To aid with the analysis and modeling of polymeric scattering data, Akcasu et al. derived an equation of motion for the time evolution of the structure factor, which can be applied to both spinodal decomposition and dissolution. We refer to this equation as the LBMA equation. There is very little literature aimed at testing the LBMA equation. To rectify this, we tested the LBMA equation using synthetic structure factor snapshots derived from simulations. In the case of dissolution, the LBMA equation performed well at describing the time evolution of the synthetic structure factor snapshots. In the case of spinodal decomposition, we determined that improvements are required. We hope our findings motivate further experimental testing and modeling work.

## Graphical abstract

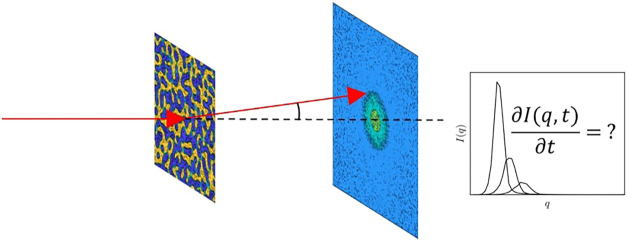

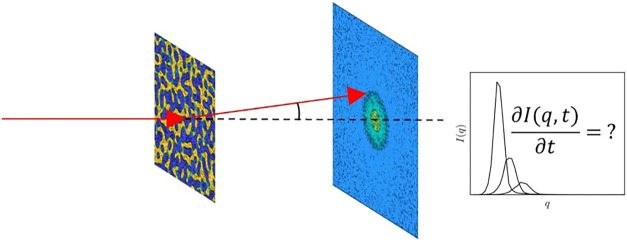

## Introduction

The fundamentals of phase separation in polymer blends^1^ attracted considerable interest in the 1980s and 1990s. This was partly because their comparatively slow dynamics, relative to liquid alloys, provided an ideal experimental platform for testing theories of phase separation. Now, there is a resurgence of interest in the phase separation of polymer blends.^2^ A central reason for this is the wide array of heterogeneous microstructures that phase separation can give rise to, which is key to the emergence of unique and desirable material properties.^3^ Notable applications of heterogeneous microstructures include organic solar cells,^4,5^ water-splitting catalysts,^6,7^ low-density high-stiffness materials,^8^ separation membranes,^9^ bone tissue engineering,^10^ and lithium-ion batteries.^11^ The process of phase separation is also attracting interest in the biological sciences since it is believed to be of relevance to a range of processes such as structural color^12^ and self-organization in biological cells.^13^

One of the challenges in developing the next generation of advanced materials, that rely on phase separation for their properties, is to be able to control, direct and arrest the phase separation.^2^ Developments in high-throughput and autonomous experimentation to increase the discovery rate of polymeric materials (see e.g., Langer et al.^14^) offer an opportunity to address this challenge. Autonomous experimentation, if enhanced with models that predict the evolution of a heterogeneous structure, could be used to develop technologies that automatically adjust processing conditions to ensure a prescribed microstructure. Since continuous measurement of the microstructure evolution of organic materials in real space is challenging, it is more likely that a technique such as small-angle scattering would be used as a noninvasive, in situ, monitoring technique,^15^ building upon its successful use in high throughput experimentation of phase separation processes in polymer mixtures.^16^ Information about the microstructure can be deduced from measurements of the structure factor - a quantity that is directly proportional to the scattered intensity.^17,18^

Thermally induced spinodal decomposition^19–21^ and dissolution^22,23^ offer a route between heterogeneous and homogeneous microstructures. During these processes, small-angle scattering provides a means of probing the evolution of the microstructure. Although the time evolution of the structure factor can be measured relatively easily, modeling it has proved to be much more difficult. The equation of motion for the structure factor during spinodal decomposition and dissolution is unclosed, i.e. an infinite hierarchy of coupled differential equations.^24–26^ As such, existing attempts to model the time evolution of the structure factor are constrained to approximate equations of motion based on truncation schemes. One such approximate equation of motion is the linear Cahn–Hilliard–Cook - Flory–Huggins–de Gennes (CHC-FHdG) equation.^27–33^ This equation has proved to be a useful tool in the analysis of scattering data.^3,19,20,34,35^ However, it is only applicable under a restrictive set of conditions and assumptions.^21,33,36^ Motivated to improve this situation, Akcasu et al. derived an approximate nonlinear equation of motion for the structure factor.^26,37,38^ Essentially, they extended the Langer, Bar-on and Miller equation^39^ for the evolution of the structure factor during the spinodal decomposition of small molecule mixtures to polymer blends by accounting for the thermodynamics associated with long-chain molecules. We refer to the resulting equation as the LBMA equation. In contrast to the linear CHC-FHdG equation, the LBMA equation has not been adopted to analyze scattering data. We believe this is because of insufficient testing. There has been no reported comparison between the predictions of the LBMA equation and numerical or experimental scattering data in the case of spinodal decomposition. In the case of dissolution, a comparison with experimental data was performed by Akcasu et al.^37^ The comparison revealed a quantitative discrepancy between theory and experiment, which worsened as the dissolution time increased. Akcasu et al. used best-guess values of the molecular and thermodynamic parameters required to solve their equation since some of the parameters are hard to measure. It is unclear whether the mismatch between theory and experiment was a result of the equation being inadequate or incorrect parameter values being used.

The LBMA equation only considers diffusive dissolution and phase separation, neglecting the role of hydrodynamics.^40^ As noted in the summary of Hashimoto’s comprehensive review^41^ and in our recent work on the wetting of nanoparticles at polymer/polymer interfaces,^42,43^ the relative importance of diffusion and hydrodynamics depends on the ratio of the dimensionless diffusion coefficient and the viscosity. The diffusion coefficient, *D* ∼ *M* ^–2^, and the viscosity, η ∼ *M* ^3.4^, scale differently with molecular weight *M*, hence the relative importance of diffusion compared to hydrodynamics on the dynamics of polymer solutions and blends strongly depends on the molecular weight. Recent modeling efforts are now starting to address what determines whether there is a transition from diffusive to hydrodynamic kinetics in polymer blends and solutions.^44^ For example, it was noted that when the phase separation also couples to vitrification, the resultant slowing down of the dynamics means that diffusion dominates the phase separation process. By comparing experimental observations with numerical modeling, Rosario-Cervellere et al.^45^ and Liu et al.^46^ have provided further evidence that a diffusion only model of phase separation provides a good qualitative description of the microstructure that evolves during polymer phase separation.

The CHC-FHdG and LBMA equations are derived from the Cahn–Hilliard–Cook (CHC) diffusion equation.^27–29^ Alternative modeling approaches to phase separation include the Lattice-Boltzmann method^47^ and the cell-dynamical approach of Shinazaki and Oono.^48^ Nonetheless, recent examples from metallurgy^49,50^ demonstrate that the phase field methodology, a broad class of models which includes the diffusive CHC equation, is a powerful foundation for predicting microstructure evolution, with the ability to *quantitatively* predict the real space growth of complex structures in metallic alloys.^49,50^ Motivated by such successes, and taking into account that for organic polymer blends, real-space, real-time microstructure monitoring is much more demanding than real-time monitoring of the structure factor in reciprocal space, in this paper, we revisit the LBMA equation of motion.

Specifically, we test the LBMA equation by comparing its numerical solution with synthetic structure factor snapshots. By using synthetic data, we remove any uncertainty in the parameter values required to solve the LBMA equation. This enables us to address whether the discrepancy between theory and experiment reported in the case of dissolution^37^ was due to incorrect parameter values or the approximations used in the derivation of the equation. We hope that our work inspires renewed efforts to develop quantitative models of microstructure evolution in polymer blends and solutions.

## Background Theory

### Polymeric Spinodal Decomposition

Let us consider an incompressible binary polymer blend. For simplicity, we assume equal degrees of polymerization, monomeric volumes, and Kuhn lengths.

Both the linear CHC-FHdG equation and the LBMA equation for the time evolution of the structure factor during polymeric spinodal decomposition and dissolution are rooted in the CHC-FHdG equation for the time evolution of the composition. We present the latter in two parts. The first part is the CHC nonlinear diffusion equation:^27–29^

1 $$\frac{\partial \phi \left(r,t\right)}{\partial t}=M\nabla^{2}\frac{\delta F\left\{\phi \left(r,t\right)\right\}}{\delta \phi }+\xi \left(r,t\right)$$

where ϕ­(***r***,*t*) is the local volume fraction (composition), *M* is the diffusional mobility, *F* is the free energy, δ/δϕ denotes a functional derivative, and ξ is a noise term. The second part is the Flory–Huggins–de Gennes free energy functional:^30,32,33,51,52^

2 $$F\left\{\phi \left(r,t\right)\right\}=\frac{k_{B}T}{v_{0}}\int d^{3}r\left(\frac{\phi }{N}\ln\left(\phi \right)+\frac{\left(1-\phi \right)}{N}\ln\left(1-\phi \right)+\chi \phi \left(1-\phi \right)+\sigma^{2}\left[\frac{1}{36}\left(\frac{1}{\phi }+\frac{1}{\left(1-\phi \right)}\right)\right]|\nabla \phi {|}^{2}\right)$$

where *k*_B_ is Boltzmann’s constant, *T* is the temperature, *v*_0_ is the monomeric volume, *N* is the degree of polymerization, σ is the Kuhn length, and χ is the interaction parameter. For simplicity, we assume a constant diffusional mobility. The coefficient to |∇ϕ|^2^ captures a cost to the free energy due to the formation of composition gradients.^27^ It is entropic in origin: gradients impose constraints on the number of configurations available to the polymers in the blend.^53^ There is also an energetic component. However, in keeping with Akcasu et al.,^26,37,38^ we have neglected this, since the entropic component often outweighs the energetic component.^32,52^ The noise term captures fluctuations in the composition arising from Brownian motion. It is modeled using a Gaussian distribution with the following moments^29,33,52,54^

3a ⟨ξ(r,t)⟩=0

3b $$\left\langle \xi \left(r,t\right)\xi \left(r^{\prime },t^{\prime }\right)\right\rangle =-2Mk_{B}T\nabla^{2}\delta \left(r-r^{\prime }\right)\delta \left(t-t^{\prime }\right)$$

The operator “–2*Mk*_B_ *T*∇^2^” ensures any change in ϕ­(***r***,*t*) due to ξ­(***r***,*t*) is balanced by the correct flux (no material is created or destroyed). It is calculated under the assumption that the noise term drives the system to the correct equilibrium state.^29,55^ Since nonlinear Langevin equations, such as eq 1, are hard to solve analytically, the most practical approach to perform this calculation is to consider the *t* → ∞ limit of the corresponding Fokker–Planck equation.^55,56^

Substituting eq 2 into eq 1 and calculating the functional derivative^33,57^ yields

4 $$\frac{\partial \phi \left(r,t\right)}{\partial t}=\frac{Mk_{B}T}{v_{0}}\nabla^{2}\left[\frac{1}{N}\ln\left(\phi \right)-\frac{1}{N}\ln\left(1-\phi \right)+\chi \left(1-2\phi \right)-\frac{\sigma^{2}}{18}\left(\frac{1}{\phi \left(1-\phi \right)}\right)\nabla^{2}\phi +\frac{\sigma^{2}}{36}\left(\frac{1-2\phi }{{\left(\phi \left(1-\phi \right)\right)}^{2}}\right){\left(\nabla \phi \right)}^{2}\right]+\xi \left(r,t\right)$$

In its current form, eq 4 can only be solved numerically.^36^ To make it analytically tractable, we must linearize it. Upon making the substitution ϕ­(***r***,*t*) = ϕ_0_ + δϕ­(***r***,*t*), where ϕ_0_ is the average volume fraction, performing a power series expansion and neglecting all nonlinear terms in δϕ­(***r***,*t*), we obtain^32,33^

5 $$\frac{\partial \delta \phi \left(r,t\right)}{\partial t}=\frac{Mk_{B}T}{v_{0}}\left[2\left(\chi_{s}-\chi \right)\nabla^{2}\delta \phi -\frac{\sigma^{2}}{18}\left(\frac{1}{\phi_{0}\left(1-\phi_{0}\right)}\right)\nabla^{4}\delta \phi \right]+\xi \left(r,t\right)$$

where χ_*s*_ = 2/(4*Nϕ*_0_(1–ϕ_0_)) is the value of the interaction parameter on the spinodal. We refer to eq 5 as the linear CHC-FHdG equation (for the composition, not the structure factor, which we present in due course). It is valid for small δϕ. Therefore, in the case of dissolution, we expect the linear CHC-FHdG equation to be valid when the composition fluctuations are small from the beginning of the process,^21,37^ which depends on how developed the initial phase-separated microstructure is. In the case of spinodal decomposition, we expect the linear CHC-FHdG equation to be valid during the early stage, i.e., when the composition fluctuations are small.^21,58^

The solution to eq 5 without noise is given by^58^

6 $$\delta \phi \left(r,t\right)=\underset{q}{\sum }\exp\left(R\left(q\right)t\right)\left(A\left(q\right)\cos\left(q\cdot r\right)+B\left(q\right)\sin\left(q\cdot r\right)\right)$$

where *A* and *B* are the initial amplitudes of the composition fluctuations present in the sample, and

7 $$R\left(q\right)=-\frac{Mk_{B}T}{v_{0}}\left[2\left(\chi_{s}-\chi \right)q^{2}+\frac{\sigma^{2}}{18}\left(\frac{1}{\phi_{0}\left(1-\phi_{0}\right)}\right)q^{4}\right]$$

is a *q*-dependent amplification factor. The symbol *q* denotes the wavenumber of a composition fluctuation, which is related to the wavelength via λ = 2π/*q*. Indeed, eq 6 suggests the morphology of a polymer blend during the early stage of spinodal decomposition can be thought of as the superposition of sinusoidal fluctuations with random orientations and amplitudes.^58^

The functional form of *R*(*q*) is shown in Figure 1 for both dissolution and spinodal decomposition. In the case of dissolution, *R*(*q*) is negative for all values of *q*, which means composition fluctuations of all wavelengths decay. In the case of spinodal decomposition, *R*(*q*) is positive for *q* < *q*_*c*_ and negative for *q* > *q*_*c*_, where *q*_*c*_ is the critical wavenumber at which *R*(*q*) = 0. This means that composition fluctuations with wavelengths λ > 2π/*q*_*c*_ will grow, while those with wavelengths λ < 2π/*q*_*c*_ will decay. Composition fluctuations with a wavelength λ_*m*_ = 2π/*q*_*m*_ will grow fastest, where *q* = *q*_*m*_ is the wavenumber at which *R*(*q*) is maximum. The wavelength λ_*m*_ corresponds to the initial value of the characteristic length scale of the blend during the early stage of spinodal decomposition.

**Figure 1:**
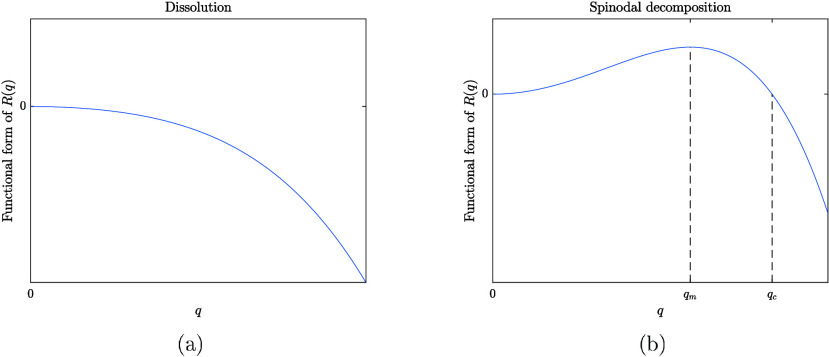
Functional form of the amplification factor, *R*(*q*), in the case of (a) dissolution and (b) spinodal decomposition. In dissolution, *R*(*q*) is negative for all values of the wavenumber *q*. In spinodal decomposition, *R*(*q*) is positive for 0 < *q* < *q*_*c*_ and negative for *q* > *q*_*c*_, where *q*_*c*_ is the critical wavenumber at which *R*(*q*) = 0. The maximum value of *R*(*q*) occurs at *q* = *q*_*m*_.

The linear CHC-FHdG equation has been shown to quantitatively capture the early stage of spinodal decomposition in polymer blends.^3,20,34,36^ Beyond the early stage, the nonlinear CHC-FHdG equation qualitatively captures the dynamics of the process.^52,58^ For example, it captures coarsening and can be used to generate simulated morphologies that are representative of spinodal decomposition.

### Small-Angle Scattering

The characteristic length scale of a blend undergoing spinodal decomposition or dissolution can be revealed by taking the Fourier transform; it corresponds to the wavenumber of the peak.^3,17,18,59^ Practically, the Fourier transform is often performed using small-angle scattering. A small-angle scattering experiment can be described in terms of three steps.^17,18^ First, radiation, typically X-rays or neutrons, is fired at a sample. Second, the radiation interacts with the sample, causing the radiation to scatter. Third, the intensity of the scattered radiation is measured as a function of the scattering angle or magnitude of the scattering vector.

The intensity of the scattered radiation is directly proportional to a quantity called the structure factor, which is essentially the power spectrum of the composition fluctuations in the blend, i.e., the product of the Fourier transform with its complex conjugate. One can learn about the microstructure by analyzing the structure factor.^3,17,18,60^ The structure factor can be written compactly as^18^

8 $$S\left(q,t\right)=\frac{1}{V}\left\langle \delta \phi \left(q,t\right)\delta \phi \left(-q,t\right)\right\rangle$$

where *q* is the magnitude of the scattering vector (***q***), *V* is the volume of the system, ⟨···⟩ denotes a time average, and δϕ­(*q*) = ∫d^3^ *r*δϕ­(***r***)­exp­(−*i* ***q*** · ***r***), i.e., δϕ­(*q*) is the Fourier transform of ϕ­(***r***) . In isotopic systems, the structure factor depends on *q* instead of ***q***. Indeed, polymer blends are generally isotropic. In an experimental context, *S*(*q*,*t*) is often calculated as the radial average of *S*(***q***,*t*).^17,52^

To aid the methodology section, we take this opportunity to introduce the power spectrum of ϕ­(***r***):

9 P(q,t)=δϕ(q,t)δϕ(−q,t)

The structure factor is related to the power spectrum via

10 $$S\left(q,t\right)=\frac{1}{V}\left\langle P\left(q,t\right)\right\rangle$$

Differentiating eq 8 with respect to time, we obtain the following equation of motion for the structure factor in terms of ∂δϕ­(*q*,*t*)/∂*t*:

11 $$\frac{\partial S\left(q,t\right)}{\partial t}=\frac{1}{V}\left\langle \frac{\partial \delta \phi \left(q\right)}{\partial t}\delta \phi \left(-q\right)\right\rangle +\frac{1}{V}\left\langle \delta \phi \left(q\right)\frac{\partial \delta \phi \left(-q\right)}{\partial t}\right\rangle$$

As shown in refs 24 and 25 , substituting the CHC-FHdG equation (eq 4) into eq 11 leads to an infinite hierarchy of coupled differential equations. Therefore, the full equation of motion for the structure factor during spinodal decomposition and dissolution is unclosed. This can be traced back to the nonlinear terms in the CHC-FHdG equation. To make the problem tractable, a truncation scheme is required.

### Linear CHC-FHdG Equation for the Structure Factor

Arguably, the simplest and most commonly used truncation scheme is based on the linear CHC-FHdG equation (eq 5),^24,25,33^ which, in Fourier space, becomes

12a $$\frac{\partial \delta \phi \left(q,t\right)}{\partial t}=-\frac{Mk_{B}Tq^{2}}{v_{0}}\left[2\left(\chi_{s}-\chi \right)+\frac{\sigma^{2}}{18\phi_{0}\left(1-\phi_{0}\right)}q^{2}\right]\phi \left(q,t\right)+\xi \left(q,t\right)$$

12b =R(q)δϕ(q,t)+ξ(q,t)

Upon substituting eq 12b and its complex conjugate into eq 11, we obtain

13 $$\frac{\partial S\left(q,t\right)}{\partial t}=2R\left(q\right)S\left(q,t\right)+\frac{1}{V}C\left(q\right)$$

where *C*(*q*) = ⟨ξ­(*q*,*t*)­δϕ­(−*q*,*t*) + ξ­(−*q*,*t*)­δϕ­(*q*,*t*)⟩. The variable *C*(*q*) appears in the definition of the covariance of the noise term in Fourier space^61^ (see reference for proof):

14 $$\left\langle \xi \left(q,t\right)\xi \left(-q,t^{\prime }\right)\right\rangle =C\left(q\right)\delta \left(t-t^{\prime }\right)$$

Therefore, *C*(*q*) can be calculated by mapping eq 3b to Fourier space:

15a $$\left\langle \xi \left(q,t\right)\xi \left(-q,t^{\prime }\right)\right\rangle =-2Mk_{B}T\delta \left(t-t^{\prime }\right)\int d^{3}r\times \int d^{3}r^{\prime }\nabla^{2}\left(\delta \left(r-r^{\prime }\right)\right)\exp\left(-iq\cdot \left(r-r^{\prime }\right)\right)$$

15b $$=-2Mk_{B}T\delta \left(t-t^{\prime }\right)\int d^{3}r\int d^{3}r^{\prime }\delta \left(r-r^{\prime }\right)\nabla^{2}\exp\left(-iq\cdot \left(r-r^{\prime }\right)\right)$$

15c $$=-2Mk_{B}T\left(-q^{2}\right)\delta \left(t-t^{\prime }\right)\int d^{3}r\int d^{3}r^{\prime }\delta \left(r-r^{\prime }\right)\exp\left(-iq\cdot \left(r-r^{\prime }\right)\right)$$

15d $$=2Mk_{B}Tq^{2}V\delta \left(t-t^{\prime }\right)$$

where we applied integration by parts twice to derive eq 15b from eq 15a. Using eq 15d, we can identify *C*(*q*) = 2*Mk*_B_ *T* *q* ^2^ *V*.

Putting everything together, we obtain the linear CHC-FHdG equation for the time evolution of the structure factor:

16 $$\frac{\partial S\left(q,t\right)}{\partial t}=2R\left(q\right)S\left(q,t\right)+2Mk_{B}Tq^{2}$$

The amplification factor can be written as^62^

17 $$R\left(q\right)=-Mk_{B}Tq^{2}S_{T}^{-1}\left(q\right)$$

where *S*_*T*_ is the stationary solution to eq 16:

18 $$S_{T}\left(q\right)=v_{0}{\left[2\left(\chi_{s}-\chi \right)+\frac{\sigma^{2}}{18}\left(\frac{1}{\phi_{0}\left(1-\phi_{0}\right)}\right)q^{2}\right]}^{-1}$$

In dissolution, *S*_*T*_ coincides with the small-*q* limit of de Gennes’ random phase approximation for the static structure factor.^62,63^ In spinodal decomposition, *S*_*T*_ coincides with the small-*q* limit of the ‘virtual’ structure factor.^62,63^ Rewriting the final term on the right-hand side of eq 16 as ‘–2*R*(*q*) *S*_*T*_(*q*)’, eq 16 can be solved using separation of variables to obtain^62^

19 $$S\left(q,t\right)=\left(S\left(q,0\right)-S_{T}\right)\exp\left(2R\left(q\right)t\right)+S_{T}\left(q\right)$$

From this solution, a fitting relationship can be established to calculate *R*(*q*) from experimental (or simulated) data.^3^ This provides a means of testing the linear CHC-FHdG equation, which, as we mentioned earlier, has been shown to quantitatively capture the early stage of spinodal decomposition in polymer blends.

### LBMA Equation for the Structure Factor

The linear CHC-FHdG equation for the structure factor is only valid during the early stage of spinodal decomposition, i.e., while composition fluctuations are small. Therefore, the validity of the linear CHC-FHdG equation is limited. Motivated to improve this situation, Akcasu et al. set out to develop an approximate, tractable, nonlinear equation of motion.^26,37,38^ Their approach is based on that of Langer, Bar-on, and Miller.^39^

To the best of our knowledge, the derivation of the LBMA equation has not been published in its entirety. In this section, we reproduce the derivation as detailed in refs 26, 37, and 38 , including additional details where appropriate.

The starting point of the derivation is the CHC-FHdG equation (eq 4). Upon writing ϕ­(***r***,*t*) = ϕ_0_ + δϕ­(***r***,*t*), performing a power series expansion up to and including third order nonlinearities, and gathering terms, we obtain

20 $$\frac{\partial \delta \phi \left(r,t\right)}{\partial t}=\frac{Mk_{B}T}{v_{0}}\nabla^{2}\left[\frac{1}{N}\ln\left(\phi_{0}\right)-\frac{1}{N}\ln\left(1-\phi_{0}\right)+\chi \left(1-2\phi_{0}\right)+\frac{\delta \phi }{N}\left(\frac{1}{\phi_{0}}+\frac{1}{1-\phi_{0}}-2N\chi \right)+\frac{\delta \phi^{2}}{N}\left(\frac{-1}{2\phi_{0}^{2}}+\frac{1}{2{\left(1-\phi_{0}\right)}^{2}}\right)+\frac{\delta \phi^{3}}{N}\left(\frac{1}{3\phi_{0}^{3}}+\frac{1}{3{\left(1-\phi_{0}\right)}^{3}}\right)-\frac{\sigma^{2}}{18}\nabla^{2}\delta \phi \left(\frac{1}{\phi_{0}}+\frac{1}{1-\phi_{0}}\right)-\frac{\sigma^{2}}{18}\left(\nabla^{2}\delta \phi \right)\times \delta \phi \left(\frac{-1}{\phi_{0}^{2}}+\frac{1}{{\left(1-\phi_{0}\right)}^{2}}\right)-\frac{\sigma^{2}}{18}\left(\nabla^{2}\delta \phi \right)\times \delta \phi^{2}\left(\frac{1}{\phi_{0}^{3}}+\frac{1}{{\left(1-\phi_{0}\right)}^{3}}\right)-\frac{\sigma^{2}}{36}{\left(\nabla \delta \phi \right)}^{2}\times \left(\frac{-1}{\phi_{0}^{2}}+\frac{1}{{\left(1-\phi_{0}\right)}^{2}}\right)-\frac{\sigma^{2}}{36}{\left(\nabla \delta \phi \right)}^{2}\times \delta \phi \left(\frac{2}{\phi_{0}^{3}}+\frac{2}{{\left(1-\phi_{0}\right)}^{3}}\right)\right]+\xi \left(r,t\right)$$

As in the linear theory (see the previous section), to derive an equation of motion for the structure factor, we must map eq 20 into Fourier space. Before doing this, it is convenient to express eq 20 in terms of the Fourier components of $\delta \phi \left(r\right)=\frac{1}{{\left(2\pi \right)}^{3}}\int d^{3}{qe}^{iq\cdot r}\delta \phi \left(q\right)$ .^64^ After simplifying, we obtain

$$\frac{\partial \delta \phi \left(r,t\right)}{\partial t}=\frac{Mk_{B}T}{v_{0}}\nabla^{2}\left[\frac{1}{{\left(2\pi \right)}^{3}N}\left(\frac{1}{\phi_{0}}+\frac{1}{1-\phi_{0}}-2N\chi \right)\times \int d^{3}q_{1}e^{iq_{1}\cdot r}\delta \phi \left(q_{1}\right)+\frac{1}{{\left(2\pi \right)}^{6}N}\left(\frac{-1}{2\phi_{0}^{2}}+\frac{1}{2{\left(1-\phi_{0}\right)}^{2}}\right)\times \underset{j=1,2}{\prod }\int d^{3}q_{j}e^{iq_{j}\cdot r}\delta \phi \left(q_{j}\right)+\frac{1}{{\left(2\pi \right)}^{9}N}\left(\frac{1}{3\phi_{0}^{3}}+\frac{1}{3{\left(1-\phi_{0}\right)}^{3}}\right)\times \underset{j=1,2,3}{\prod }\int d^{3}q_{j}e^{iq_{j}\cdot r}\delta \phi \left(q_{j}\right)+\frac{\sigma^{2}}{18{\left(2\pi \right)}^{3}}\left(\frac{1}{\phi_{0}}+\frac{1}{1-\phi_{0}}\right)\times \int d^{3}q_{1}q_{1}^{2}e^{iq_{1}\cdot r}\delta \phi \left(q_{1}\right)+\frac{\sigma^{2}}{18{\left(2\pi \right)}^{6}}\left(\frac{-1}{\phi_{0}^{2}}+\frac{1}{{\left(1-\phi_{0}\right)}^{2}}\right)\times \int d^{3}q_{1}q_{1}^{2}e^{iq_{1}\cdot r}\delta \phi \left(q_{1}\right)\int d^{3}q_{2}e^{iq_{2}\cdot r}\delta \phi \left(q_{2}\right)+\frac{\sigma^{2}}{18{\left(2\pi \right)}^{9}}\left(\frac{1}{\phi_{0}^{3}}+\frac{1}{{\left(1-\phi_{0}\right)}^{3}}\right)\int d^{3}q_{1}q_{1}^{2}e^{iq_{1}\cdot r}\delta \phi \left(q_{1}\right)\times \underset{j=2,3}{\prod }\int d^{3}q_{j}e^{iq_{j}\cdot r}\delta \phi \left(q_{j}\right)+\frac{\sigma^{2}}{36{\left(2\pi \right)}^{6}}\left(\frac{-1}{\phi_{0}^{2}}+\frac{1}{{\left(1-\phi_{0}\right)}^{2}}\right)\times \int d^{3}q_{1}q_{1}e^{iq_{1}\cdot r}\delta \phi \left(q_{1}\right)\cdot \int d^{3}q_{2}q_{2}e^{iq_{2}\cdot r}\delta \phi \left(q_{2}\right)+\frac{\sigma^{2}}{36{\left(2\pi \right)}^{9}}\left(\frac{2}{\phi_{0}^{3}}+\frac{2}{{\left(1-\phi_{0}\right)}^{3}}\right)\int d^{3}q_{1}q_{1}e^{iq_{1}\cdot r}\delta \phi \left(q_{1}\right),\cdot \int d^{3}q_{2}q_{2}e^{iq_{2}\cdot r}\delta \phi \left(q_{2}\right)\int d^{3}q_{3}e^{iq_{3}\cdot r}\delta \phi \left(q_{3}\right)\right]+\xi \left(r,t\right)$$ 21

Next, mapping eq 21 into Fourier space, making use of the result ∫d^3^ *r* *e* ^(*i*(∑_*j*_ ***q***_*j*_)· ***r***)^ = (2π)^3^δ­(∑_*j*_ ***q***_*j*_),^64^ and simplifying, yields

22 $$\frac{\partial \delta \phi \left(q,t\right)}{\partial t}=\frac{-Mk_{B}Tq^{2}}{v_{0}}\left[\delta \phi \left(q\right)\left\{\frac{1}{N}\left(\frac{1}{\phi_{0}}+\frac{1}{1-\phi_{0}}-2N\chi \right)+\frac{\sigma^{2}}{18}\left(\frac{1}{\phi_{0}}+\frac{1}{1-\phi_{0}}\right)q^{2}\right\}+\frac{1}{{\left(2\pi \right)}^{3}}\times \left(\int d^{3}q_{1}\int d^{3}q_{2}\delta \left(q-q_{1}-q_{2}\right)\delta \phi \left(q_{1}\right)\delta \phi \left(q_{2}\right)\Gamma_{2}\left(q_{1},q_{2}\right)\right)+\frac{1}{{\left(2\pi \right)}^{6}}\left(\int d^{3}q_{1}\int d^{3}q_{2}\int d^{3}q_{3}\delta \left(q-q_{1}-q_{2}-q_{3}\right)\delta \phi \left(q_{1}\right)\times \delta \phi \left(q_{2}\right)\delta \phi \left(q_{3}\right)\Gamma_{3}\left(q_{1},q_{2}\right)\right)\right]+\xi \left(q,t\right)$$

where we defined the following vertex functions:

23a $$\Gamma_{2}\left(q_{1},q_{2}\right)=\frac{1}{N}\left(\frac{-1}{2\phi_{0}^{2}}+\frac{1}{2{\left(1-\phi_{0}\right)}^{2}}\right)+\frac{\sigma^{2}}{18}\left(\frac{-1}{\phi_{0}^{2}}+\frac{1}{{\left(1-\phi_{0}\right)}^{2}}\right)q_{1}^{2}+\frac{\sigma^{2}}{36}\left(\frac{-1}{\phi_{0}^{2}}+\frac{1}{{\left(1-\phi_{0}\right)}^{2}}\right)q_{1}\cdot q_{2}$$

23b $$\Gamma_{3}\left(q_{1},q_{2}\right)=\frac{1}{N}\left(\frac{1}{3\phi_{0}^{3}}+\frac{1}{3{\left(1-\phi_{0}\right)}^{3}}\right)+\frac{\sigma^{2}}{18}\left(\frac{1}{\phi_{0}^{3}}+\frac{1}{{\left(1-\phi_{0}\right)}^{3}}\right)q_{1}^{2}+\frac{\sigma^{2}}{36}\left(\frac{2}{\phi_{0}^{3}}+\frac{2}{{\left(1-\phi_{0}\right)}^{3}}\right)q_{1}\cdot q_{2}$$

The vertex functions capture the coupling between composition fluctuations with different wavevectors, i.e. the coupling between different fluctuation modes. As it stands, Γ_2_ and Γ_3_ are incomplete. The vertex function Γ_2_ should capture the coupling between pairs of fluctuation modes with wavevectors that add up to ***q***. As such, Γ_2_ must be a symmetric function of ***q***_1_ and ***q***_2_. Similarly, the vertex function Γ_3_ should capture the coupling between triplets of fluctuation modes with wavevectors that add up to ***q***. As such, Γ_3_ must be a symmetric function of ***q***_1_, ***q***_2_ and ***q***_3_. The complete vertex functions are the average over all possible permutational pairs of the relevant wavevectors. For example, in the case of Γ_2_:

24 $$\Gamma_{2}\left(q_{1},q_{2}\right)=\frac{1}{N}\left(\frac{-1}{2\phi_{0}^{2}}+\frac{1}{2{\left(1-\phi_{0}\right)}^{2}}\right)+\frac{\sigma^{2}}{36}\left(\frac{-1}{\phi_{0}^{2}}+\frac{1}{{\left(1-\phi_{0}\right)}^{2}}\right)\times \left[q_{1}^{2}+q_{2}^{2}+\frac{1}{2}\left(q_{1}\cdot q_{2}+q_{2}\cdot q_{1}\right)\right]$$

Setting ***q*** = ***q***_1_ + ***q***_2_ and making the substitution ***q***_1_· ***q***_2_ + ***q***_2_· ***q***_1_ = *q* ^2^ – *q*_1_^2^ – *q*_2_^2^ yields

25 $$\Gamma_{2}\left(q,q_{1},q_{2}\right)=\frac{1}{N}\left(\frac{-1}{2\phi_{0}^{2}}+\frac{1}{2{\left(1-\phi_{0}\right)}^{2}}\right)+\frac{\sigma^{2}}{72}\left(\frac{-1}{\phi_{0}^{2}}+\frac{1}{{\left(1-\phi_{0}\right)}^{2}}\right)\times \left[q^{2}+q_{1}^{2}+q_{2}^{2}\right]$$

Similarly, in the case of Γ_3_(***q***, ***q***_1_, ***q***_2_, ***q***_3_), we obtain

26 $$\Gamma_{3}\left(q,q_{1},q_{2},q_{3}\right)=\frac{1}{N}\left(\frac{1}{3\phi_{0}^{3}}+\frac{1}{3{\left(1-\phi_{0}\right)}^{3}}\right)+\frac{\sigma^{2}}{108}\left(\frac{1}{\phi_{0}^{3}}+\frac{1}{{\left(1-\phi_{0}\right)}^{3}}\right)\times \left[q^{2}+q_{1}^{2}+q_{2}^{2}+q_{3}^{2}\right]$$

In terms of these complete, symmetric vertex functions, eq 22 becomes

27 $$\frac{\partial \delta \phi \left(q,t\right)}{\partial t}=R\left(q\right)\delta \phi \left(q\right)-\frac{Mk_{B}Tq^{2}}{v_{0}{\left(2\pi \right)}^{3}}\int d^{3}q_{1}\times \int d^{3}q_{2}\delta \left(q-q_{1}-q_{2}\right)\delta \phi \left(q_{1}\right)\delta \phi \left(q_{2}\right)\times \Gamma_{2}\left(q,q_{1},q_{2}\right)-\frac{Mk_{B}Tq^{2}}{v_{0}{\left(2\pi \right)}^{6}}\int d^{3}q_{1}\int d^{3}q_{2}\times \int d^{3}q_{3}\delta \left(q-q_{1}-q_{2}-q_{3}\right)\delta \phi \left(q_{1}\right)\delta \phi \left(q_{2}\right)\times \delta \phi \left(q_{3}\right)\Gamma_{3}\left(q,q_{1},q_{2},q_{3}\right)+\xi \left(q,t\right)$$

where we identified the coefficient of δϕ­(***q***) as the amplification factor, *R*(*q*) (eq 7). Substituting eq 27 and its complex conjugate into eq 11 yields the following equation of motion for the structure factor

28 $$\frac{\partial S\left(q,t\right)}{\partial t}=2\left[R\left(q\right)S\left(q,t\right)-\frac{Mk_{B}Tq^{2}}{v_{0}V{\left(2\pi \right)}^{3}}\int d^{3}q_{1}\times \int d^{3}q_{2}\delta \left(q-q_{1}-q_{2}\right)\left\langle \delta \phi \left(q_{1}\right)\delta \phi \left(q_{2}\right)\delta \phi \left(-q\right)\right\rangle \Gamma_{2}\left(q,q_{1},q_{2}\right)-\frac{Mk_{BT}q^{2}}{v_{0}V{\left(2\pi \right)}^{6}}\int d^{3}q_{1}\int d^{3}q_{2}\int d^{3}q_{3}\delta \left(q-q_{1}-q_{2}-q_{3}\right)\times \left\langle \delta \phi \left(q_{1}\right)\delta \phi \left(q_{2}\right)\delta \phi \left(q_{3}\right)\delta \phi \left(-q\right)\right\rangle \Gamma_{3}\left(q,q_{1},q_{2},q_{3}\right)\right]+\frac{1}{V}\left\langle \xi \left(q,t\right)\delta \phi \left(-q\right)\right\rangle +\frac{1}{V}\left\langle \xi \left(-q,t\right)\delta \phi \left(q\right)\right\rangle$$

Equation 28 is not closed since the third- and fourth-order correlation functions (the second and third terms in the square brackets, respectively) cannot be written in terms of *S*(***q***,*t*) . To obtain a tractable equation of motion for the structure factor, Akcasu et al. approximated the composition fluctuations in Fourier space as a Gaussian process with zero mean. Making use of Isserlis’ theorem,^65^ the third-order correlation function can then be set equal to zero and the fourth-order correlation function can be written in terms of the second-order correlation function, i.e., *S*(***q***,*t*) . Specifically, making use of the permutational symmetry of the integrals in eq 28:

29a $$\left\langle \delta \phi \left(q_{1}\right)\delta \phi \left(q_{2}\right)\delta \phi \left(-q\right)\right\rangle =0$$

29b $$\left\langle \delta \phi \left(q_{1}\right)\delta \phi \left(q_{2}\right)\delta \phi \left(q_{3}\right)\delta \phi \left(-q\right)\right\rangle =3{\left(2\pi \right)}^{6}S\left(q,t\right)S\left(q_{2},t\right)\delta \left(q-q_{1}\right)\times \delta \left(q_{2}+q_{3}\right)$$

Substituting eqs 29a and 29b into eq 28 yields

$$\frac{\partial S\left(q,t\right)}{\partial t}=2\left[R\left(q\right)S\left(q,t\right)-\frac{3S\left(q,t\right)Mk_{B}Tq^{2}}{v_{0}V}\int d^{3}q_{1}\int d^{3}q_{2}\times \int d^{3}q_{3}\delta \left(q-q_{1}-q_{2}-q_{3}\right)S\left(q_{2},t\right)\delta \left(q-q_{1}\right)\delta \left(q_{2}+q_{3}\right),\Gamma_{3}\times \left(q,q_{1},q_{2},q_{3}\right)\right]+\frac{1}{V}C\left(q\right)$$ 30

Care is required at this stage. Since the blend in question has a finite volume, the integrals should technically be replaced with sums, i.e., ∫d^3^ *q*_1_ → ((2π)^3^/*V*)∑_***q***_1__ and so on.^66^ Along with this change, the delta functions must be replaced with Kronecker deltas, i.e., δ­(***q*** – ***q***_1_ – ***q***_2_ – ***q***_3_) → (*V*/(2π)^3^) δ_***q***,__***q***_1_ + ***q***_2_ + ***q***_3__ and so on.^66^ It follows that, in the finite volume limit, eq 30 becomes

31 $$\frac{\partial S\left(q,t\right)}{\partial t}=2\left[R\left(q\right)S\left(q,t\right)-\frac{3S\left(q,t\right)Mk_{B}Tq^{2}}{v_{0}V}\times \underset{q_{1}}{\sum }\underset{q_{2}}{\sum }\underset{q_{3}}{\sum }\delta_{q,q_{1}+q_{2}+q_{3}}S\left(q_{2},t\right)\delta_{q,q_{1}}\delta_{q_{2},-q_{3}}\Gamma_{3}\left(q,q_{1},q_{2},q_{3}\right)\right]+\frac{1}{V}C\left(q\right)$$

Upon carrying out the summations, making use of the Kronecker deltas, we obtain

32 $$\frac{\partial S\left(q,t\right)}{\partial t}=2R\left(q\right)S\left(q,t\right)\left[1-\frac{3Mk_{B}Tq^{2}}{v_{0}VR\left(q\right)}\underset{q_{2}}{\sum }S\left(q_{2},t\right)\Gamma_{3}\left(q,q_{2}\right)\right]+\frac{1}{V}C\left(q\right)$$

where Γ_3_(***q***, ***q***_2_) = (1/*N*)­(1/(3ϕ_0_^3^) + 1/(3­(1–ϕ_0_)^3^)) + (σ^2^/108)­(1/(ϕ_0_^3^) + 1/(1–ϕ_0_)^3^)­(2*q* ^2^ + 2*q*_2_^2^). Equation 32 can be written more compactly as

33 $$\frac{\partial S\left(q,t\right)}{\partial t}=2R\left(q\right)S\left(q,t\right)\left[1+Z\left(q,t\right)\right]+\frac{1}{V}C\left(q\right)$$

where we have made use of the fact that that polymer blends are, in general, isotropic by writing *S*(***q***,*t*) as *S*(*q*,*t*) . The product *R*(*q*)­[1 + *Z*(*q*,*t*)] can be thought of as a modified amplification factor.

To determine *C*(*q*), we consider the *t* → ∞ limit of eq 33, requiring that *S*(*q*,*t*) → *S*_eq_(*q*), hence

34 $$C\left(q\right)=-2VR\left(q\right)S_{\mathrm{eq}}\left(q\right)\left[1+Z_{\mathrm{eq}}\left(q\right)\right]$$

which allows us to calculate *C*(*q*) in terms of *S*_eq_(*q*) consistently with the closure approximations. The equilibrium structure factor must be specified by independent calculations. At this point in the derivation, we must specialize to either dissolution or spinodal decomposition.

#### Dissolution

In dissolution, *S*_eq_(*q*) can be set equal to de Gennes’ random phase approximation for the static structure factor. In the small-*q* limit, this is given by eq 18. With an equation for *S*_eq_(*q*), we can proceed with the calculation of *Z*_eq_(*q*) to complete the specification of the noise term. We start with the equation for *Z*(*q*,*t*), which, in full, is given by

35 $$Z\left(q,t\right)=\frac{-Mk_{B}Tq^{2}}{{\left(2\pi \right)}^{3}v_{0}R\left(q\right)}\left(\frac{1}{\phi_{0}^{3}}+\frac{1}{{\left(1-\phi_{0}\right)}^{3}}\right)\left\{\left(\frac{1}{N}+\frac{\sigma^{2}q^{2}}{18}\right)\int d^{3}q_{2}S\left(q_{2},t\right)+\frac{\sigma^{2}}{18}\int d^{3}q_{2}q_{2}^{2}S\left(q_{2},t\right)\right\}$$

where, for convenience, we have reverted back to the infinite volume limit (∑_***q*** 2_ → (*V*/(2π)^3^)∫d^3^ *q*_2_). Writing the integrals in terms of spherical polar coordinates and simplifying yields

36 $$Z\left(q,t\right)=\frac{-Mk_{B}Tq^{2}}{2\pi^{2}v_{0}R\left(q\right)}\left(\frac{1}{\phi_{0}^{3}}+\frac{1}{{\left(1-\phi_{0}\right)}^{3}}\right)\left\{\left(\frac{1}{N}+\frac{\sigma^{2}q^{2}}{18}\right)\times \int_{0}^{q_{\mathrm{cut}}}dq_{2}q_{2}^{2}S\left(q_{2},t\right)+\frac{\sigma^{2}}{18}\int_{0}^{q_{\mathrm{cut}}}dq_{2}q_{2}^{4}S\left(q_{2},t\right)\right\}$$

where the upper limit *q*_cut_ is introduced to ensure the integral is convergent. The physical significance of *q*_cut_ is that only fluctuation modes with wavenumbers between 0 and *q*_cut_ are coupled. Akcasu et al. advocate setting *q*_cut_ ≈ *q*_*c*_, where *q*_*c*_ is the inverse correlation length

37 $$q_{c}=\sqrt{\frac{36\left(\chi_{s}-\chi \right)\left(\phi_{0}\left(1-\phi_{0}\right)\right)}{\sigma^{2}}}$$

The inverse correlation length is defined by expressing eq 18 in the form *S*_eq_(*q*) = *S*(0)/(1 + (*q*/*q_*c*_*)^2^). Akcasu et al. introduced the parameter α = *q*_cut_/*q*_*c*_ as a measure of the range of mode coupling. This is treated as an adjustable parameter in the theory. Rewriting the integrals in eq 36 in terms of *q̃* = *q*/*q*_*c*_ yields

38 $$Z\left(q,t\right)=\frac{-Mk_{B}Tq^{2}q_{c}^{3}}{2\pi^{2}v_{0}R\left(q\right)}\left(\frac{1}{\phi_{0}^{3}}+\frac{1}{{\left(1-\phi_{0}\right)}^{3}}\right)\left\{\left(\frac{1}{N}+\frac{\sigma^{2}q^{2}}{18}\right)\times \int_{0}^{\alpha }d{\mathrm{q̃}}_{2}{\mathrm{q̃}}_{2}^{2}S\left({\mathrm{q̃}}_{2}q_{c},t\right)+\frac{\sigma^{2}q_{c}^{2}}{18}\int_{0}^{\alpha }d{\mathrm{q̃}}_{2}{\mathrm{q̃}}_{2}^{4}S\left({\mathrm{q̃}}_{2}q_{c},t\right)\right\}$$

In the limit *t* → ∞, replacing *S*(*q*,*t*) with *S*_eq_(*q*), we obtain

39 $$Z_{\mathrm{eq}}\left(q\right)=\frac{-Mk_{B}Tq^{2}q_{c}^{3}}{2\pi^{2}R\left(q\right)}\left(\frac{1}{\phi_{0}^{3}}+\frac{1}{{\left(1-\phi_{0}\right)}^{3}}\right){\left(2\chi_{s}-2\chi \right)}^{-1}\times \left\{\left(\frac{1}{N}+\frac{\sigma^{2}q^{2}}{18}\right)\int_{0}^{\alpha }d{\mathrm{q̃}}_{2}{\mathrm{q̃}}_{2}^{2}{\left(1+{\mathrm{q̃}}_{2}^{2}\right)}^{-1}+\frac{\sigma^{2}q_{c}^{2}}{18}\int_{0}^{\alpha }d{\mathrm{q̃}}_{2}{\mathrm{q̃}}_{2}^{4}{\left(1+{\mathrm{q̃}}_{2}^{2}\right)}^{-1}\right\}$$

Upon evaluating the integrals, we arrive at

40 $$Z_{\mathrm{eq}}\left(q\right)=\frac{-Mk_{B}Tq^{2}q_{c}^{3}}{2\pi^{2}R\left(q\right)}\left(\frac{1}{\phi_{0}^{3}}+\frac{1}{{\left(1-\phi_{0}\right)}^{3}}\right){\left(2\chi_{s}-2\chi \right)}^{-1}\times \left\{\left(\frac{1}{N}+\frac{\sigma^{2}q^{2}}{18}\right)\left(\alpha -\tan^{-1}\left(\alpha \right)\right)+\frac{\sigma^{2}q_{c}^{2}}{18}\times \left(\frac{1}{3}\alpha^{3}-\alpha +\tan^{-1}\left(\alpha \right)\right)\right\}$$

Substituting eqs 7, 18, and 40 into eq 34, the noise term in the case of dissolution is given by

41 $$C\left(q\right)=2Mk_{B}TVq^{2}\left[1+\frac{q_{c}^{3}v_{0}}{8\pi^{2}}\left(\frac{1}{\phi_{0}^{3}}+\frac{1}{{\left(1-\phi_{0}\right)}^{3}}\right){\left(\chi_{s}-\chi \right)}^{-2}\times {\left(1+{\left(\frac{q}{q_{c}}\right)}^{2}\right)}^{-1}\left\{\left(\frac{1}{N}+\frac{\sigma^{2}q^{2}}{18}\right)\left(\alpha -\tan^{-1}\left(\alpha \right)\right)+\frac{\sigma^{2}q_{c}^{2}}{18}\left(\frac{1}{3}\alpha^{3}-\alpha +\tan^{-1}\left(\alpha \right)\right)\right\}\right]$$

where we made use of eq 17.

Putting everything together, the LBMA equation applied to dissolution is given by eq 33, where *R*(*q*) is given by eq 7, *Z*(*q*,*t*) is given by eq 38, and *C*(*q*) is given by eq 41.

The linear CHC-FHdG equation applied to dissolution is recovered from the LBMA equation when we set *Z*(*q*,*t*) = *Z*_eq_(*q*) = 0.

#### Spinodal Decomposition

In the case of spinodal decomposition, Akcasu et al. argued that the static structure factor at the final temperature is unknown,^67,68^ meaning eq 34 cannot be applied. They suggested a workaround for this in the case of small changes in χ, i.e., small temperature jumps. Namely, assuming we know the value of χ in the one-phase region before the initiation of spinodal decomposition, denoted χ_*i*_, we could make the approximation *C*(*q*,χ_*f*_) ≈ *C*(*q*,χ_*i*_), where χ_*f*_ is the value of χ inside the spinodal.

Since χ = χ_*f*_ > χ_*s*_ in spinodal decomposition, we must define the inverse correlation length in a different way to eq 37. Following Akcasu et al., we define

42 $$q_{c,2}=\sqrt{\frac{36\left(\chi -\chi_{s}\right)\left(\phi_{0}\left(1-\phi_{0}\right)\right)}{\sigma^{2}}}$$

Putting everything together, the LBMA equation applied to spinodal decomposition is given by eq 33, where *R*(*q*) is given by eq 7 with χ = χ_*f*_, *Z*(*q*,*t*) is given by eq 38, with *q*_*c*_ = *q*_*c*,2_ and χ = χ_*f*_, and *C*(*q*) is given by eq 41 with χ = χ_*i*_.

The linear CHC-FHdG equation applied to spinodal decomposition is recovered from the LBMA equation when we set *Z*(*q*,*t*) = *Z*_eq_(*q*) = 0.

### The Small-*q* Limit

Both the linear CHC-FHdG equation and the LBMA equation (eqs 16 and 33, respectively) are only valid in the small-*q* limit. This is a consequence of substituting the FHdG free energy functional (eq 2) into the CHC equation (eq 1). The FHdG free energy functional was derived to be consistent with the small-*q* limit of de Gennes’ random phase approximation for the static structure factor (see Section S.1.3 of the Supporting Information for more details).^53,62–64^ The small-*q* (or “long wavelength”) limit is defined as *qR*_g_ ≪ 1,^62,64^ where *R*_g_ is a representative radius of gyration for the polymers in the blend.

## Methodology

### Generating Time Series of Synthetic Structure Factor Snapshots

#### Overview and Key Equations

We used two time series of synthetic structure factor snapshots to obtain our results. An overview of the method we used to generate each time series of synthetic structure factor snapshots is as follows:

- We simulated polymeric spinodal decomposition or dissolution using a finite difference scheme, namely, a nondimensionalised and discretized version of the CHC-FHdG equation (eq 4).
- During the simulations, we calculated snapshots of the power spectrum using a modified version of eq 9.
- After running several repeat simulations, we calculated snapshots of the structure factor by applying a modified version of eq 10 to the power spectrum snapshots we accumulated.

There are three key equations: the finite difference scheme and the equations we used to calculate the snapshots of the power spectrum and the structure factor. We present these equations below. In the interest of orderliness, we defer the derivations of the equations to Section S.1.1 of the Supporting Information.

#### Finite Difference Scheme

To simulate polymeric spinodal decomposition and dissolution, we applied the following finite difference scheme – a nondimensionalised and discretized version of eq 4 – to a simple cubic lattice with periodic boundary conditions:

43 $$\phi_{j,k,l}^{m+1}=\phi_{j,k,l}^{m}+\frac{\Delta \tau }{2\Delta x^{2}}\underset{nn}{\sum }\left[\frac{\chi_{c}}{2\left|\chi -\chi_{s}\right|}\ln\left(\frac{\phi_{j,k,l}^{m}}{1-\phi_{j,k,l}^{m}}\right)-\frac{2\chi \phi_{j,k,l}^{m}}{\left|\chi -\chi_{s}\right|}+\frac{1}{36}\left(\frac{1-2\phi_{j,k,l}^{m}}{{\left(\phi_{j,k,l}^{m}\left(1-\phi_{j,k,l}^{m}\right)\right)}^{2}}\right)\frac{1}{4\Delta x^{2}}\underset{nn}{\prod }\phi_{j,k,l}^{m}-2\left(\frac{1}{36\phi_{j,k,l}^{m}\left(1-\phi_{j,k,l}^{m}\right)}\right)\frac{1}{\Delta x^{2}}\underset{nn}{\sum }\phi_{j,k,l}^{m}\right]+\frac{v_{0}^{1/2}|\chi -\chi_{s}{|}^{1/4}}{\sigma^{3/2}}\frac{1}{\Delta x}\left[\eta_{1;j+1,k,l}^{m}-\eta_{1;j,k,l}^{m}+\eta_{2;j,k+1,l}^{m}-\eta_{2;j,k,l}^{m}+\eta_{3;j,k,l+1}^{m}-\eta_{3;j,k,l}^{m}\right]$$

where *m* denotes the number of dimensionless time steps, Δτ is the duration of a dimensionless time step, *j*, *k*, and *l* denote the coordinates of each lattice site, Δ*x* is the dimensionless length of each lattice site, ∑_nn_ and ∏_nn_ are short-hand operators, and η_1_, η_2_, and η_3_ are dimensionless Gaussian random variables. The shorthand operators are defined as

44a $$\underset{nn}{\sum }f_{j,k,l}=f_{j+1,k,l}+f_{j-1,k,l}+f_{j,k+1,l}+f_{j,k-1,l}+f_{j,k,l+1}+f_{j,k,l-1}-6f_{j,k,l}$$

44b $$\underset{nn}{\prod }f_{j,k,l}=f_{j+1,k,l}^{2}+f_{j-1,k,l}^{2}+f_{j,k+1,l}^{2}+f_{j,k-1,l}^{2}+f_{j,k,l+1}^{2}+f_{j,k,l-1}^{2}-2\left(f_{j+1,k,l}f_{j-1,k,l}+f_{j,k+1,l}f_{j,k-1,l}+f_{j,k,l+1}f_{j,k,l-1}\right)$$

The first and second moments of the Gaussian random variables are given by

45a $$\left\langle \eta_{n;j,k,l}^{m}\right\rangle =0$$

45b $$\left\langle \eta_{n;j,k,l}^{m}\eta_{n\prime ;j\prime ,k\prime ,l\prime }^{m\prime }\right\rangle =\frac{\Delta \tau }{\Delta x^{3}}\delta_{n,n\prime }\delta_{j,j\prime }\delta_{k,k\prime }\delta_{l,l\prime }\delta_{m,m\prime }$$

The dimensionless variables we used to obtain eq 43 are as follows:

46a $$x=\frac{|\chi -\chi_{s}{|}^{1/2}}{\sigma }r$$

46b $$\tau =\frac{2Mk_{B}T|\chi -\chi_{s}{|}^{2}}{\sigma^{2}v_{0}}t$$

46c $$\mathrm{\xi ̃}\left(x,\tau \right)=\frac{\sigma^{2}v_{0}}{2Mk_{B}T|\chi -\chi_{s}{|}^{2}}\xi \left(r,t\right)$$

These dimensionless variables are inspired by those in ref 52. They relate to the fastest growing wavelength and its growth rate during the early stage of spinodal decomposition.

In the context of lattice sites in the top row of a cubic lattice, periodic boundary conditions mean that the nearest neighbors in the vertical direction are the corresponding lattice sites in the bottom row. The nearest neighbors to the left of lattice sites in the left-most row, to the right of lattice sites in the right-most row, and below lattice sites in the bottom row follow analogously.

For later reference, we denote the total number of time steps in a simulation as *m*_max_.

#### Snapshots of the Power Spectrum

To calculate snapshots of the power spectrum during the simulations, we used

$${\mathrm{P̃}}_{n;d}^{m}=\Delta x^{6}\langle \overset{N_{s}-1}{\underset{j=0}{\sum }}\overset{N_{s}-1}{\underset{k=0}{\sum }}\overset{N_{s}-1}{\underset{l=0}{\sum }}\delta \phi_{n;j,k,l}^{m}e^{-\frac{2\pi i}{N_{s}}(\mathrm{aj}+bk)}\times \overset{N_{s}-1}{\underset{j^{\prime }=0}{\sum }}\overset{N_{s}-1}{\underset{k^{\prime }=0}{\sum }}\overset{N_{s}-1}{\underset{l^{\prime }=0}{\sum }}\delta \phi_{n;j\prime ,k\prime ,l\prime }^{m\prime }e^{\frac{2\pi i}{N_{s}}\left(aj\prime +bk\prime \right)}\rangle_{R}$$ 47

where *n* allows one to distinguish between repeat simulations, *N*_*s*_ is the number of lattice sites in each dimension of the cubic simulation lattice, *a* and *b* are integers in the range −(*N*_*s*_ – 1)/2 ≤ *a*,*b* ≤ (*N*_*s*_ – 1)/2, and ⟨···⟩_*R*_ denotes a radial average. The radial average can be written explicitly as

48 $$\langle f_{a,b}\rangle_{R}\equiv f_{d}=\frac{\underset{a,b\;\mathrm{s.t.}\;\mathrm{round}\left(\sqrt{a^{2}+b^{2}}\right)=d}{\sum }f_{a,b}}{\underset{a,b\;\mathrm{s.t.}\;\mathrm{round}\left(\sqrt{a^{2}+b^{2}}\right)=d}{\sum }1}$$

where *d* is an integer in the range 0 ≤ *d* ≤ (*N*_*s*_ – 1)/2.

#### Snapshots of the Structure Factor

After implementing *N*_*r*_ repeat simulations, we calculated snapshots of the structure factor using

49 $${\mathrm{S̃}}_{d}^{m}=\frac{1}{N_{r}N_{s}^{3}\Delta x^{3}}\overset{N_{r}}{\underset{n=1}{\sum }}{\mathrm{P̃}}_{n;d}^{m}$$

Throughout the remainder of the paper, we express snapshots of *S̃*_*d*_^*m*^ as *S̃*(k,τ), where k = 2*d*π/(*N*_*s*_Δ*x*) and τ = *m*Δτ. The symbol k denotes the magnitude of the dimensionless scattering vector, i.e., k = |**k**|.

We note that eqs 47 and 49 are nondimensional and discrete, consistent with eq 43. The nondimensionalisation was performed using eq 46a and the dimensionless variables below:

50a $$k=\frac{\sigma }{|\chi -\chi_{s}{|}^{1/2}}q$$

50b $$\mathrm{S̃}\left(k,\tau \right)=\frac{|\chi -\chi_{s}{|}^{3/2}}{\sigma^{3}}S\left(q,t\right)$$

#### Parameter Values and Initial Conditions

Each of the time series we generated corresponds to different χ values. For ease of reference, we devised a name for each time series based on these values - see Table 1. There are two values of χ listed for the dissolution time series because of how we generated the initial composition when simulating dissolution - we discuss this below. We implemented five repeat simulations (*N*_*r*_ = 5) to generate each time series. During the simulations, we calculated snapshots of the power spectrum after the first and every 400th time step, i.e., when *m* = 1, 400, 800, 1200, etc. The values of the other parameters we used in the simulations were Δ*x* = 0.25, Δ*τ* = 6.25 × 10^–5^, ϕ_0_ = 0.5, *N*_*s*_ = 257, *m*_max_ = 8 × 10^5^, *N* = 2700, and $\sigma =\sqrt{20}v_{0}^{1/3}$ , where the factor of √20 is the square root of the characteristic ratio, $C_{\infty }=\sigma^{2}/v_{0}^{2/3}$ . For both time series, the value of χ_*s*_ corresponding to *N* = 2700 is χ_*s*_ = 0.000741. We based the values of *N* and χ on those used in refs. 69–71 The value of *C*_∞_ corresponds to a relatively stiff polymer^72^ - we found using larger values of *C*_∞_ increased the stability of the simulations. We used trial and error to determine suitable values of Δ*x* and Δ*τ*, i.e., values of Δ*x* and Δ*τ* that can be used in the simulations to generate time series that are independent of these values. Further details on this are provided in Section S.1.2 of the Supporting Information.

**Table 1:** Value(s) of *χ* Corresponding to Each Time Series We Generated

time series name	χ
dissolution	0.000765 → 0.000716
spinodal decomposition	0.000765

In the simulations of spinodal decomposition, we set the initial composition at each lattice site equal to ϕ_0_. After the first time step, the Gaussian random variable term in eq 43 introduced random fluctuations into the composition, initiating spinodal decomposition. In the simulations of dissolution, we generated the initial composition, i.e. the composition at the time dissolution is initiated, by simulating spinodal decomposition with ϕ_0_ = 0.5 and χ = 0.000765 for the first 1.6 × 10^5^ time steps. We then initiated dissolution by making a step change to χ = 0.000716. The functions of |χ – χ_*s*_| in eq 43 are a result of the nondimensionalisation. To avoid the scaling between the dimensionless and real variables changing between the two stages of the dissolution simulations, we chose the initial and final values of χ such that |0.000765 – χ_*s*_| = |χ_*s*_ – 0.000716|.

#### Implementing the Simulations

We implemented the simulations in Julia.^73^ To keep the computation time to a minimum, we made use of the CUDA.jl package.^74^ This allowed us to implement eq 43 on a graphics processing unit (GPU), which we accessed on the University of Sheffield’s high-performance computer, Stanage. The code we developed to implement the simulations is available on ORDA, the University of Sheffield’s research data repository. Text files from which the time series can be calculated using eq 49 are also located there. Please see the data availability statement at the end of the paper for more details.

#### Conforming with the Small-k Limit

As we have already mentioned, eq 43 is nondimensionalised and discretized version of eq 4. Since eq 4 is only valid in the small-*q* limit, it follows that eq 43 is only valid in the corresponding small-k limit. The small-k limit is somewhat ambiguously defined as k*r*_*g*_ ≪ 1, where *r*_*g*_ is the dimensionless radius of gyration. To determine whether to truncate the snapshots of the synthetic structure factor we generated to conform with the small-k limit when obtaining our results, we attempted to quantify the small-k limit.

We determined inequalities that specify the small-k limit corresponding to each time series. We then compared these inequalities with the k-values associated with the constituent snapshots. For both time series, we determined the small-k limit corresponds to *k* < 5 (the scaling to *q* is given by eq 50a). We outline the calculations we performed to determine this inequality in Section S.1.3 of the Supporting Information. Given that we used *N*_*s*_ = 257 and Δ*x* = 0.25 in the simulations, each snapshot of the synthetic structure factor we generated consists of 129 values, with the 129th value corresponding to k ≈ 12.5. Therefore, we concluded that truncating the snapshots is necessary to conform with the small-k limit.

### Testing the LBMA equation

To test the LBMA equation applied to dissolution, we compared its numerical solution with synthetic structure factor snapshots from the dissolution time series and the numerical solution to the linear CHC-FHdG equation applied to dissolution. To test the LBMA equation applied to spinodal decomposition, we compared its numerical solution with synthetic structure factor snapshots from the spinodal decomposition time series and the numerical solution to the linear CHC-FHdG equation applied to spinodal decomposition.

#### Dimensionless Equations

In order to compare their numerical solutions with the synthetic structure factor snapshots, we substituted the dimensionless variables in eqs 46b, 50a, and 50b into the aforementioned equations.

#### Dissolution

The dimensionless LBMA equation applied to dissolution is given by

51 $$\frac{\partial \mathrm{S̃}\left(k,\tau \right)}{\partial \tau }=2r\left(k\right)\mathrm{S̃}\left(k,\tau \right)\left[1+Z\left(k,\tau \right)\right]+C\left(k\right)$$

where

52a $$r\left(k\right)=\frac{-k^{2}}{\left|\chi -\chi_{s}\right|}\left(\left(\chi_{s}-\chi \right)+\frac{\left|\chi -\chi_{s}\right|k^{2}}{36\phi_{0}\left(1-\phi_{0}\right)}\right)$$

52b $$Z\left(k,\tau \right)=\frac{-k^{2}}{4\pi^{2}\left|\chi -\chi_{s}\right|r\left(k\right)}\left(\frac{1}{\phi_{0}^{3}}+\frac{1}{{\left(1-\phi_{0}\right)}^{3}}\right)\times \left\{\left(\frac{1}{N}+\frac{\left|\chi -\chi_{s}\right|}{18}k^{2}\right)\int_{0}^{k_{\mathrm{cut}}}d{\mathrm{kk}}^{2}\mathrm{S̃}\left(k,\tau \right)+\frac{\left|\chi -\chi_{s}\right|}{18}\int_{0}^{k_{\mathrm{cut}}}d{\mathrm{kk}}^{4}\mathrm{S̃}\left(k,\tau \right)\right\}$$

52c $$C\left(k\right)=\frac{v_{0}|\chi -\chi_{s}{|}^{1/2}k^{2}}{\sigma^{3}}\left[1+\frac{v_{0}q_{c}^{3}}{8\pi^{2}{\left(\chi_{s}-\chi \right)}^{2}}\left(\frac{1}{\phi_{0}^{3}}+\frac{1}{{\left(1-\phi_{0}\right)}^{3}}\right)\times {\left(1+\frac{\left|\chi -\chi_{s}\right|k^{2}}{36\left(\chi_{s}-\chi \right)\phi_{0}\left(1-\phi_{0}\right)}\right)}^{-1}\left\{\left(\frac{1}{N}+\frac{\left|\chi -\chi_{s}\right|}{18}k^{2}\right)\times \left(\alpha -\tan^{-1}\left(\alpha \right)\right)+\frac{\sigma^{2}q_{c}^{2}}{18}\left(\frac{1}{3}\alpha^{3}-\alpha +\tan^{-1}\left(\alpha \right)\right)\right\}\right]$$

The upper limit in the integrals is given by *k*_cut_ = αk_*c*_, where k_*c*_ is related to *q*_*c*_ via eq 50a.

In the dimensionless linear CHC-FHdG equation applied to dissolution, *r*(k) is given by eq 52a, *Z*(k,τ) = 0 and *C*(k) is given by eq 52c, but with the second term, i.e., the term after the plus sign inside the square brackets, set equal to zero.

#### Spinodal Decomposition

In the dimensionless LBMA equation applied to spinodal decomposition, *r*(k), *Z*(k,τ) and *C*(k) are given by

53a $$r\left(k\right)=\frac{-k^{2}}{\left|\chi -\chi_{s}\right|}\left(\left(\chi_{s}-\chi_{f}\right)+\frac{\left|\chi -\chi_{s}\right|k^{2}}{36\phi_{0}\left(1-\phi_{0}\right)}\right)$$

53b $$Z\left(k,\tau \right)=\frac{-k^{2}}{4\pi^{2}\left|\chi -\chi_{s}\right|r\left(k\right)}\left(\frac{1}{\phi_{0}^{3}}+\frac{1}{{\left(1-\phi_{0}\right)}^{3}}\right)\left\{\left(\frac{1}{N}+\frac{\left|\chi -\chi_{s}\right|}{18}k^{2}\right)\times \int_{0}^{k_{\mathrm{cut}}}d{\mathrm{kk}}^{2}\mathrm{S̃}\left(k,\tau \right)+\frac{\left|\chi -\chi_{s}\right|}{18}\int_{0}^{k_{\mathrm{cut}}}d{\mathrm{kk}}^{4}\mathrm{S̃}\left(k,\tau \right)\right\}$$

53c $$C\left(k\right)=\frac{v_{0}|\chi -\chi_{s}{|}^{1/2}k^{2}}{\sigma^{3}}\left[1+\frac{v_{0}q_{c}^{3}}{8\pi^{2}{\left(\chi_{s}-\chi_{i}\right)}^{2}}\left(\frac{1}{\phi_{0}^{3}}+\frac{1}{{\left(1-\phi_{0}\right)}^{3}}\right)\times {\left(1+\frac{\left|\chi -\chi_{s}\right|k^{2}}{36\left(\chi_{s}-\chi_{i}\right)\phi_{0}\left(1-\phi_{0}\right)}\right)}^{-1}\left\{\left(\frac{1}{N}+\frac{\left|\chi -\chi_{s}\right|}{18}k^{2}\right)\times \left(\alpha -\tan^{-1}\left(\alpha \right)\right)+\frac{\sigma^{2}q_{c}^{2}}{18}\left(\frac{1}{3}\alpha^{3}-\alpha +\tan^{-1}\left(\alpha \right)\right)\right\}\right]$$

The upper limit in the integrals is given by *k*_cut_ = α *k*_c, 2_, where *k*_c, 2_ is related to *q*_*c*,2_ via eq 50a.

In the dimensionless linear CHC-FHdG equation applied to spinodal decomposition, *r*(k) is given by eq 53a, *Z*(k,τ) = 0 and *C*(k) is given by eq 53c, but with the second term set equal to zero.

#### Solving the Dimensionless Equations

To calculate the numerical solutions to the dimensionless equations, we made approximations consistent with those used to obtain and solve eq 43. Namely, we approximated continuous time as a series of discrete time steps of duration Δτ and the continuous wavenumber as k = 2dπ/(*N*Δ*x*), where *d* is an integer in the range 0 ≤ *d* ≤ (*N*_*s*_ – 1)/2. We approximated the time derivative as a forward finite difference scheme and calculated the integrals using the trapezoidal method. The numerical solutions to the resulting discretized equations were calculated by integrating both sides of the equations over a single time step, approximating the integration as a Riemann sum with a single term. We ensured the numerical solutions were independent of the value of Δτ.

We set *N* = 2700, ϕ_0_ = 0.5 and $\sigma =\sqrt{20}v_{0}^{1/3}$ , and we investigated three different values of α: 0.5, 1 and 1.5.

In the equations corresponding to dissolution, we set χ = 0.000716. The initial condition was the snapshot corresponding to τ = 10 in the dissolution time series. We note that in the simulations of dissolution used to generate the dissolution time series, dissolution was initiated at τ = 10.

In the equations corresponding to spinodal decomposition, we set χ_*i*_ = 0.000716 and χ_*f*_ = χ = 0.000765. The initial condition was the snapshot corresponding to τ = 6.25 × 10^–5^ in the spinodal decomposition time series, i.e., the first snapshot in the time series.

## Results and Analysis

### Dissolution

We now present our findings from testing the LBMA equation applied to dissolution. Figure 2 compares synthetic structure factor snapshots from the dissolution time series with the numerical solutions to the dimensionless LBMA equation and the dimensionless linear CHC-FHdG equation. Three solutions to the LBMA equation were calculated, corresponding to α = 0.5, 1, and 1.5. These values of α relate to *k*_cut_ = 1.5, *k*_cut_ = 3 and *k*_cut_ = 4.5, respectively.

**Figure 2:**
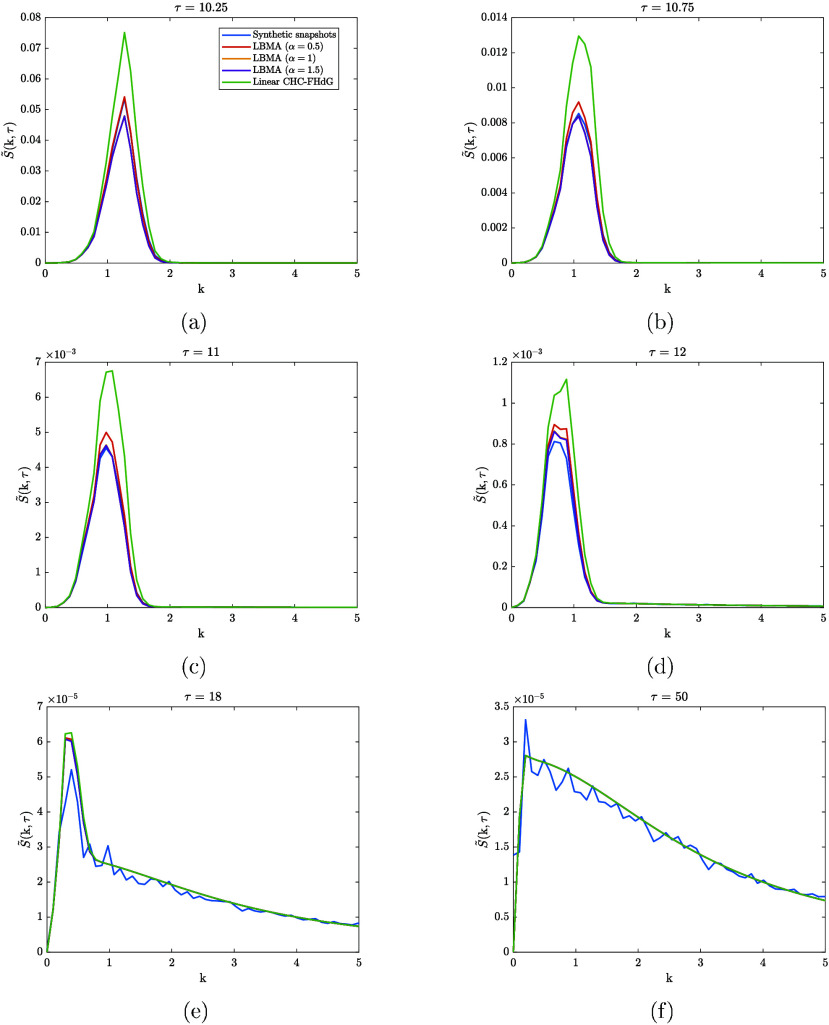
A comparison between synthetic structure factor snapshots from the dissolution time series and the numerical solutions to the dimensionless LBMA equation applied to dissolution and the linear CHC-FHdG equation applied to dissolution. Three solutions to the LBMA equation were calculated, corresponding to α = 0.5, 1, and 1.5. These values of α relate to k_cut_ = 1.5, k_cut_ = 3 and k_cut_ = 4.5, respectively. We note that in the simulations of dissolution used to generate the dissolution time series, dissolution was initiated at τ = 10. The data corresponds to times that lag behind the onset of dissolution by 0.25, 0.75, 1, 2, 8, and 40 dimensionless time units, respectively.

The LBMA equation describes the time evolution of the synthetic structure factor snapshots more accurately than the linear CHC-FHdG equation for 10 < τ < 18: there is more overlap between the synthetic structure factor snapshots and the solutions to the LBMA equation than there is between the synthetic structure factor snapshots and the solution to the linear CHC-FHdG equation. For τ ≥ 18, the solutions to the LBMA equation more or less coincide with the solution to the linear CHC-FHdG equation. We infer that the increased accuracy of the LBMA equation compared to the linear CHC-FHdG equation for 10 < τ < 18 is a result of the mode-coupling term, *Z*(k,τ), in the former, which is the distinguishing feature between the two equations.

Mode-coupling affects the rate at which the solutions to the LBMA equation grow and/or decay: in eq 51, the product *r*(k)­[1 + Z­(k,τ)] can be thought of as a modified amplification factor. Figure 3 compares the time evolution of the modified amplification factors associated with the different solutions in Figure 2. There is only a short period for which the different amplification curves do not overlap. This suggests there is only a short period for which mode-coupling has an appreciable effect on the dynamics predicted by the LBMA equation. The idea that mode-coupling becomes less appreciable during dissolution is consistent with the fact that dissolution causes the magnitudes of the composition fluctuations to decrease. As can be verified using Figure 2, the effect of mode-coupling is to enhance the decay rate of the solutions to the LBMA equation, and, up to α = 1, the decay rate increases with α. The latter of these findings suggests that, during the period that mode-coupling is appreciable, only the coupling between modes with 0 < k < k_*c*_ (k_*c*_ = 3) affects the decay rate.

**Figure 3:**
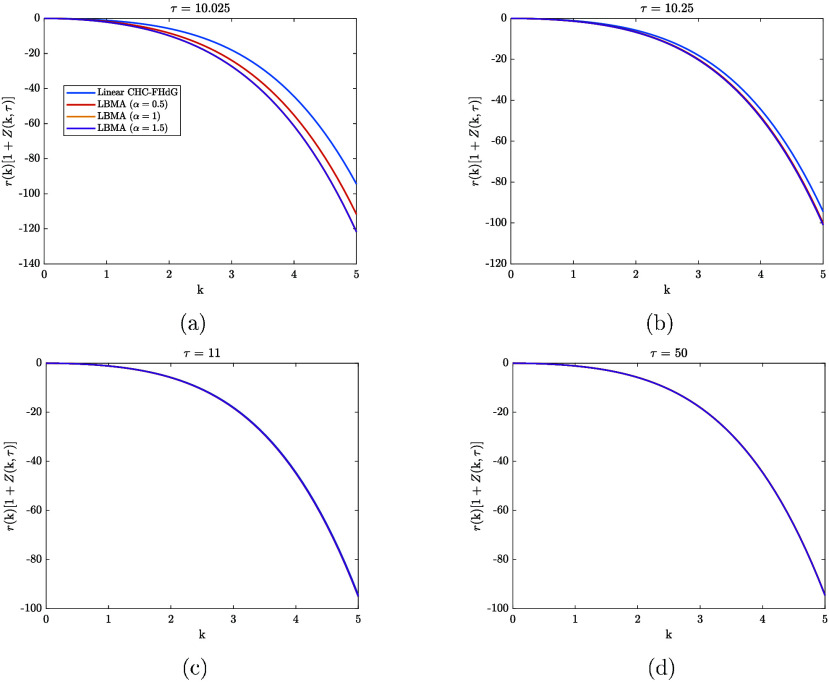
Comparison between the amplification factors associated with the different solutions in Figure 2 at various values of τ.

Turning our attention back to Figure 2, we can see that, around τ = 12, the three solutions to the LBMA equation coincide. Later on, around τ = 18, these solutions coincide with the solution to the linear CHC-FHdG equation. We think the most probable explanation for this phenomenon is that the noise term, *C*(k), begins to dominate the dynamics predicted by the LBMA equation. Consider the equation that results from substituting eq 34 into eq 33. As the first term on the right-hand side approaches the second term on the right-hand side, the rate of change of the solution will decrease. Based on the data in Figures 2 and 3, we propose that this will happen earlier for larger values of α, allowing the curves corresponding to the other solutions to ‘catch up’. In this context, we can think of the linear CHC-FHdG equation as the LBMA equation in which α = 0.

We believe the idea that the noise begins to dominate the dynamics predicted by the LBMA equation might also explain the temporary discrepancy that develops between the peaks of the synthetic structure factor snapshots and those of the solutions to the LBMA equation around τ = 12. We are not entirely sure how we could reduce this artifact. Including more mode-coupling terms in the LBMA equation seems unlikely to help. This would mean including more terms in the power series expansion in eq 20. We expect higher-order terms can be ignored as τ increases.

Both the LBMA equation and the linear CHC-FHdG equation accurately predict the locations of the peaks of the synthetic structure factor snapshots, i.e., k_*m*_(τ) . Furthermore, as expected, both the LBMA equation and the linear CHC-FHdG equation capture the correct equilibrium behavior.

The LBMA equation performed much better here, when tested using synthetic structure factor snapshots, than it did when tested using experimental scattering data by Akcasu et al.^37^ Since the parameter values corresponding to the synthetic structure factor snapshots are known, this suggests that part of the reason for the experimental discrepancy could be poor estimates of the hard-to-measure thermodynamic and molecular parameters.

To end this section, we provide an in-depth analysis of the time evolution of *k*_m_ and *S̃*(*k*_m_,τ), which we hope might be of experimental interest. First, we focus on the time evolution of *k*_m_, which we determined via two methods: (1) using the synthetic structure factor snapshots from the dissolution time series, and (2) using the numerical solution to the LBMA equation applied to dissolution (with α = 1). Figure 4 shows our results. As expected from Figure 2, the LBMA data agrees well with the synthetic data. Akcasu et al. proposed that a *k*_m_ ∼ *t* ^–1/2^ power law may be briefly observed, prior to thermal fluctuations becoming significant, when the initial state is prepared via spinodal decomposition.^26^ This is consistent with the power law dependence of the diffusion length from Fick’s second law. This power law is included in Figure 4, from which it can be seen that both the synthetic and LBMA data approximately follow the power law for a brief period.

**Figure 4:**
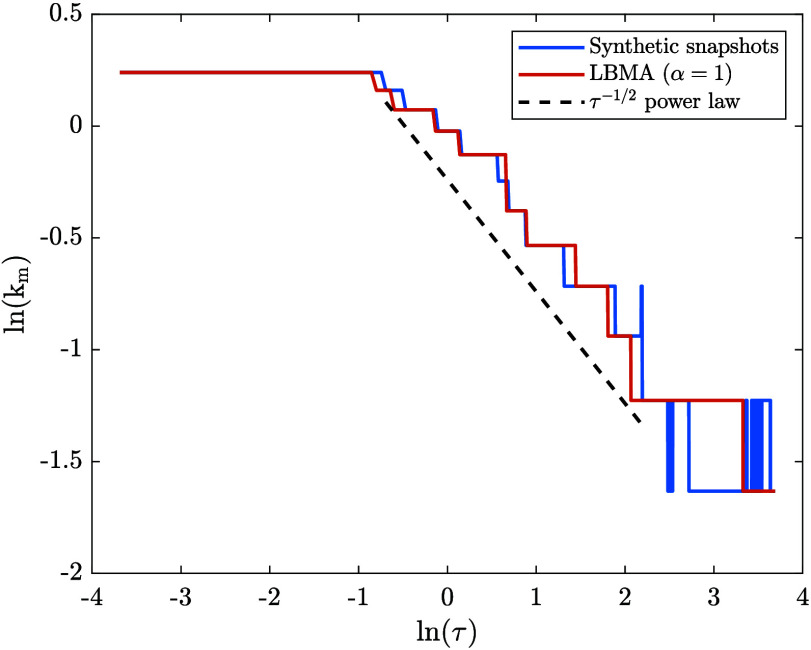
Comparison between the time evolution of *k*_m_ during dissolution obtained via two methods: (1) using the synthetic structure factor snapshots from the dissolution time series, and (2) using the numerical solution to the LBMA equation applied to dissolution (with α = 1).

In Figure 5, we show the time evolution of *S̃*(*k*_m_,τ), which, as expected from Figure 2, reveals a good agreement between the LBMA data and the synthetic data.

**Figure 5:**
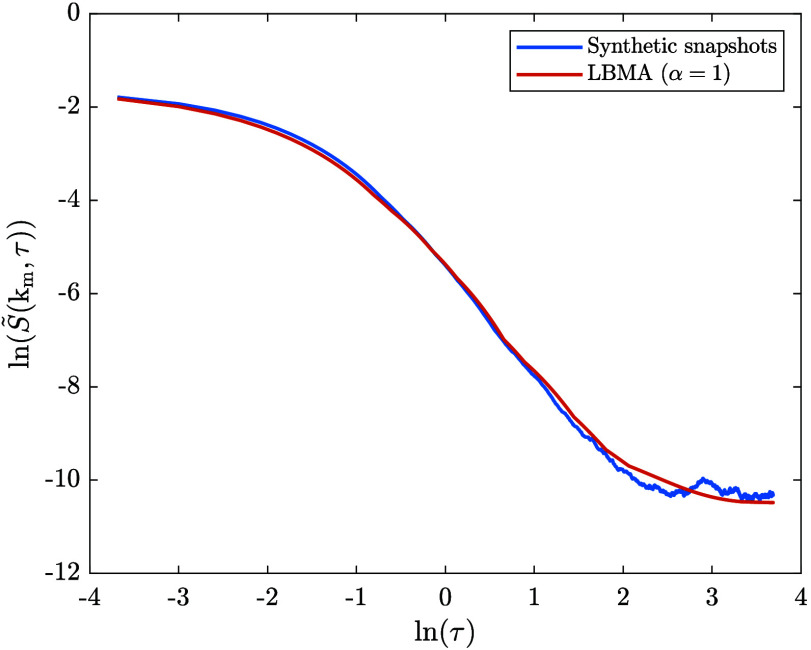
Comparison of the time evolution of *S̃*(*k*_m_,τ) during spinodal decomposition, obtained via two methods: (1) using the synthetic structure factor snapshots from the dissolution time series, and (2) using the numerical solution to the LBMA equation applied to dissolution (with α = 1).

### Spinodal Decomposition

Next, we present our findings from testing the LBMA equation applied to spinodal decomposition. Figure 6 compares synthetic structure factor snapshots from the spinodal decomposition time series with the numerical solutions to the dimensionless LBMA equation and the dimensionless linear CHC-FHdG equation. Three solutions to the LBMA equation were calculated, corresponding to α = 0.5, 1, and 1.5. These values of α relate to k_cut_ = 1.5, k_cut_ = 3 and k_cut_ = 4.5, respectively. For τ > 5, the solution to the linear CHC-FHdG equation is not plotted since it grows exponentially.

**Figure 6:**
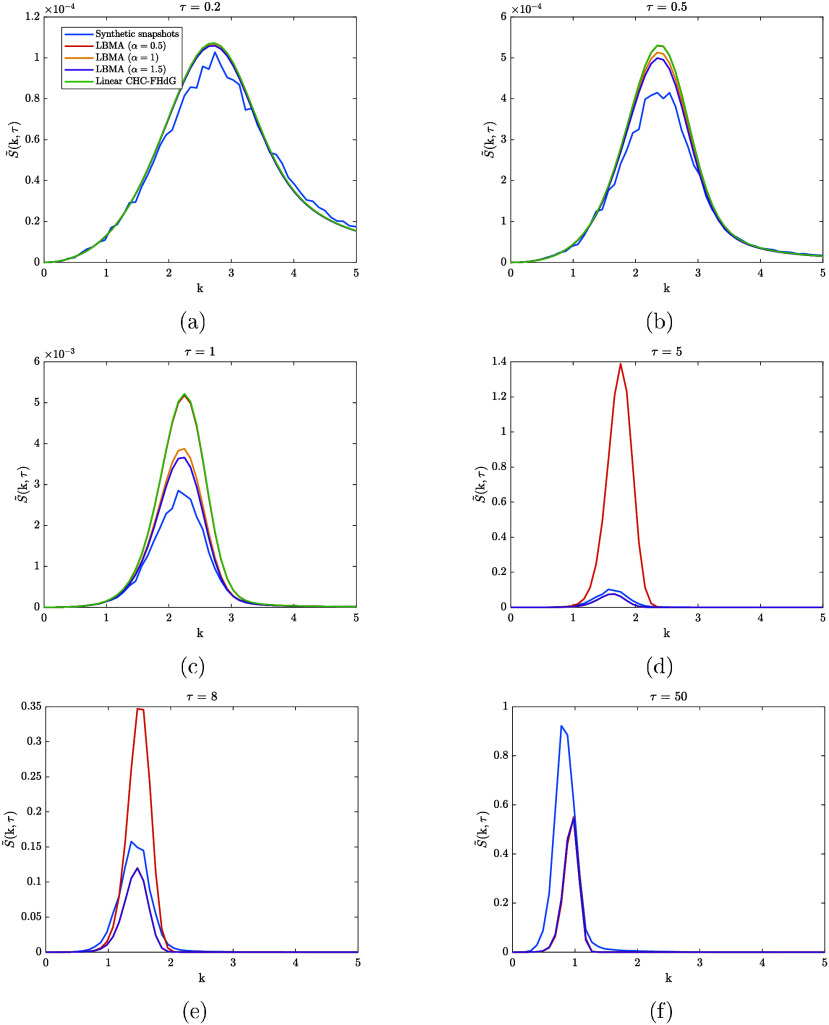
Comparison between synthetic structure factor snapshots from the spinodal decomposition time series and the numerical solutions to the dimensionless LBMA equation applied to spinodal decomposition and the linear CHC-FHdG equation applied to spinodal decomposition. Three solutions to the LBMA equation were calculated, corresponding to α = 0.5, 1, and 1.5. These values of α relate to k_cut_ = 1.5, k_cut_ = 3 and k_cut_ = 4.5, respectively. For τ > 5, the solution to the linear CHC-FHdG equation is not plotted since it grows exponentially.

During the early stage of spinodal decomposition, i.e. up to about τ = 0.2, both the LBMA equation and the linear CHC-FHdG equation describe the time evolution of the synthetic structure factor snapshots fairly accurately. As expected in this regime, the LBMA equation reduces to the linear CHC-FHdG equation: the solutions to both equations overlap. Just after τ = 0.2, the solutions to both equations start to diverge from one another, and both equations overpredict the synthetic structure factor snapshots, but, in the cases where it was solved with α = 1 and α = 1.5, the LBMA equation does so less dramatically. We infer that this improvement of the LBMA equation over the linear CHC-FHdG equation is rooted in the mode-coupling term of the former. At a time τ > 1, the solutions to the LBMA equation calculated with α = 1 and α = 1.5 coincide and begin to under-predict the synthetic structure factor snapshots. Later on, these solutions coincide with the solution calculated with α = 0.5. Interestingly, the curves corresponding to the α = 0.5 solution decrease and move toward the curves corresponding to the α = 1 and α = 1.5 solutions. While we believe this behavior to be unphysical, it can be explained by considering the modified amplification factor.

Figure 7 compares the time evolution of the modified amplification factors associated with the different solutions in Figure 6. The *x*-axis is truncated at π to aid the distinguishability of the amplification curves. For a while after the onset of spinodal decomposition, the amplification curves corresponding to the α = 0.5 solution to the LBMA equation overlap with the amplification curves corresponding to the solution of the linear CHC-FHdG equation, i.e., *r*(k) . This suggests that, during this period, the coupling between modes with 0 < k < 0.5k_*c*_ (k_*c*_ = 3) has no appreciable effect on the dynamics predicted by the LBMA equation. Contrary to this, the amplification curves corresponding to the α = 1 and α = 1.5 solutions to the LBMA equation shift below *r*(k) . As can be verified using Figure 6, the effect of the mode-coupling is to slow the growth rate of the α = 1 and α = 1.5 solutions to the LBMA equation. Furthermore, we note that the mode coupling enhances the decay rate of these solutions at larger k-values. Around τ = 5, the amplification curve corresponding to the α = 0.5 solution to the LBMA equation shifts below those corresponding to the α = 1 and α = 1.5 solutions, with the former having a larger range of negative k-values (associated with decay). Based on eq 53b (*Z*(k,τ) depends on *S̃*(k,τ)), we believe the reason for this shift is that the initial unconstrained growth of the α = 0.5 solution to the LBMA equation eventually leads to appreciable mode-coupling between modes with 0 < k < 0.5k_*c*_. As a result, the α = 0.5 solution to the LBMA equation tends toward the α = 1 and α = 1.5 solutions, eventually coinciding. As this happens, the amplification curve corresponding to the α = 0.5 solution to the LBMA equation shifts toward and eventually coincides with the amplification curves corresponding to the α = 1 and α = 1.5 solutions.

**Figure 7:**
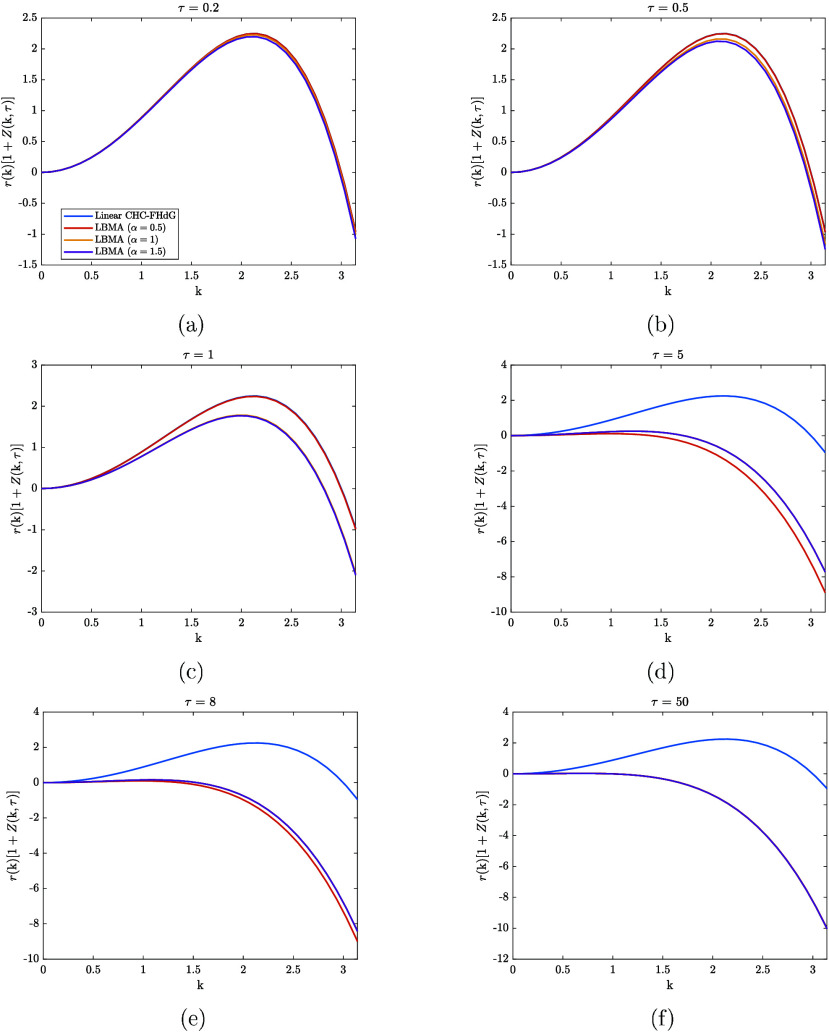
Comparison between the amplification factors associated with the different solutions in Figure 6 at various values of τ. The *x*-axis is truncated at π to aid the distinguishability of the amplification curves.

The fact that the different solutions to the LBMA equation end up coinciding (as do the corresponding amplification curves) implies that the range of k-values for which the effects of mode-coupling are appreciable on the dynamics predicted by the LBMA equation shifts to 0 < k < 0.5k_*c*_. This is consistent with coarsening, which causes *k*_*m*_(τ) to shift to smaller values. Indeed, the LBMA equation captures this effect of coarsening fairly accurately - a well-known pitfall of the linear CHC-FHdG equation.

Unlike in the case of dissolution, the noise term does not begin to dominate the dynamics of the solutions to the LBMA equation as τ increases. Instead, the solutions to the LBMA equation continue to grow, albeit at a slower rate than the synthetic structure factor snapshots. We believe this slower growth rate to be a consequence of mode-coupling. It seems that the approximation *C*(k,χ_*f*_) ≈ *C*(k,χ_*i*_) under the assumption of a small temperature jump does not apply to the spinodal decomposition time series.

As we did in the previous section, we end this section with an in-depth analysis of the time evolution of k_m_ and *S̃*(k_m_,τ) . First, we focus on the time evolution of *k*_m_, which we determined via two methods: (1) using the synthetic structure factor snapshots from the spinodal decomposition time series, and (2) using the numerical solution to the LBMA equation applied to spinodal decomposition (with α = 1). Figure 8 shows our results. As expected from Figure 6, the LBMA data agrees fairly well with the synthetic data, with a slight discrepancy at later times. Since the spinodal decomposition time series was generated using the diffusive Cahn–Hilliard–Cook Flory–Huggins–de Gennes equation, a power law of k_m_ ∼ *t* ^–1/3^ is expected at late times (see, for example, refs 71 and 75 ). This power law is included in Figure 8, from which it can be seen that the synthetic data roughly follows the power law at later times, although the LBMA data appears to follow a power law with a slightly smaller exponent. This is consistent with the findings reported by Akcasu et al.^38^

**Figure 8:**
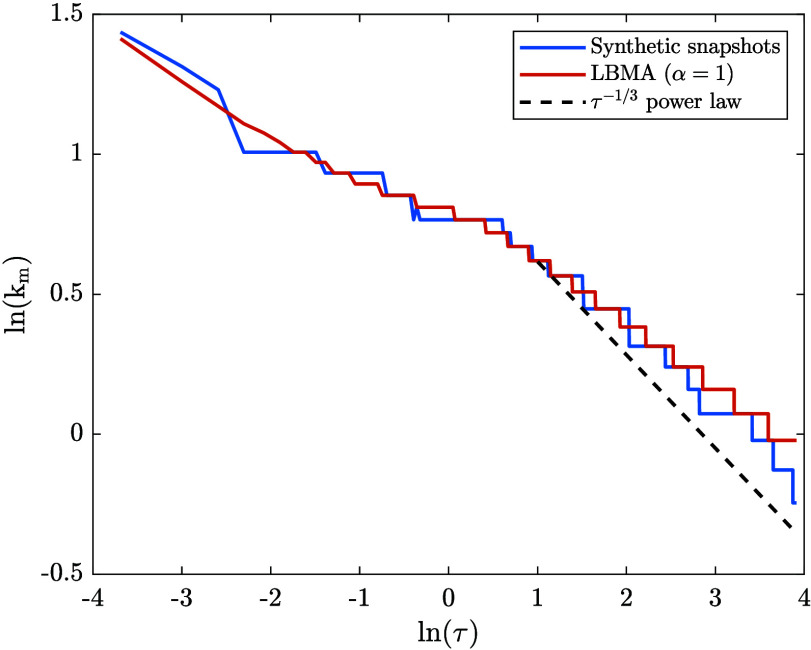
Comparison between the time evolution of k_m_ during spinodal decomposition obtained via two methods: (1) using the synthetic structure factor snapshots from the spinodal decomposition time series, and (2) using the numerical solution to the LBMA equation applied to spinodal decomposition (with α = 1).

In Figure 9, we show the time evolution of *S̃*(k_max_,τ), which, as expected from Figure 6, reveals a good agreement between the synthetic and LBMA data only during the early stage of spinodal decomposition. As a result of the dynamical scaling hypothesis, the synthetic data is expected to scale as *S̃*(k,τ) = k_m_^–3^ *F*(k/k_m_) ∼ τ^1^ at late times. This power law is included in Figure 9 from which it can be seen that the synthetic data approximately follows the power law. As expected, the LBMA data follows a power law with a slightly smaller exponent.

**Figure 9:**
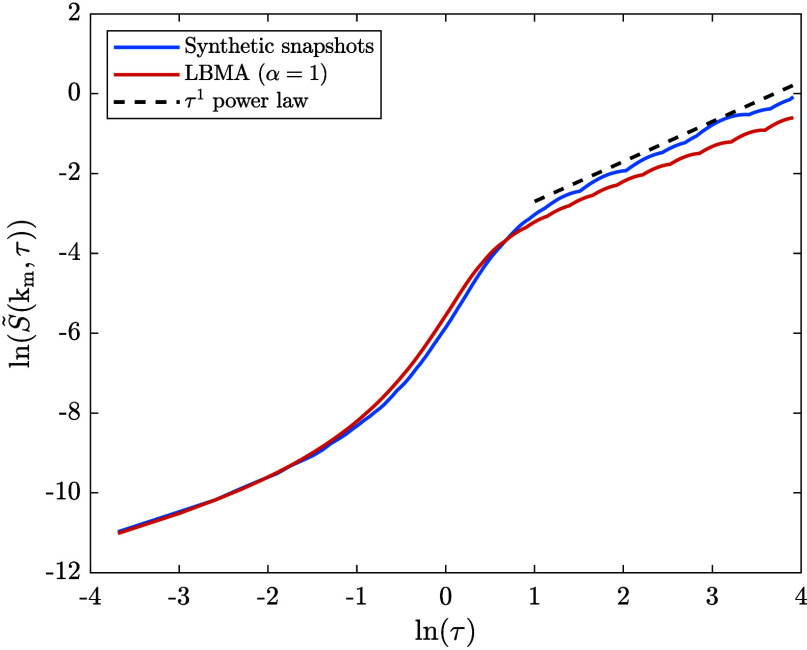
Comparison between the time evolution of *S̃*(k_m_,τ) during spinodal decomposition obtained via two methods: (1) using the synthetic structure factor snapshots from the spinodal decomposition time series, and (2) using the numerical solution to the LBMA equation applied to spinodal decomposition (with α = 1).

### The ‘Asymmetry’ Between the Dynamics of Spinodal Decomposition and Dissolution

To tie up our in-depth analyses of the time evolution of k_m_ and *S̃*(k_m_) provided at the end of the last two sections, we briefly turn our attention to the asymmetry between the rate at which composition fluctuations grow during spinodal decomposition and decay during dissolution. While this observation could be inferred from the figures above, it is perhaps most apparent when the time evolution of *S̃*(k_m_,τ) during a sequential process; i.e., spinodal decomposition immediately followed by dissolution, is plotted, as shown in Figure 10. The synthetic data in the figure was derived from the dissolution time series, which contains spinodal decomposition structure factor snapshots up to τ = 10 followed by dissolution snapshots up to τ = 50. The LBMA data in the figure comes from the numerical solutions to the LBMA equation applied to spinodal decomposition and dissolution, respectively. To solve the former, the initial condition was the first snapshot in the dissolution time series. To solve the latter, the initial condition was the snapshot corresponding to τ = 10 in the dissolution time series.

**Figure 10:**
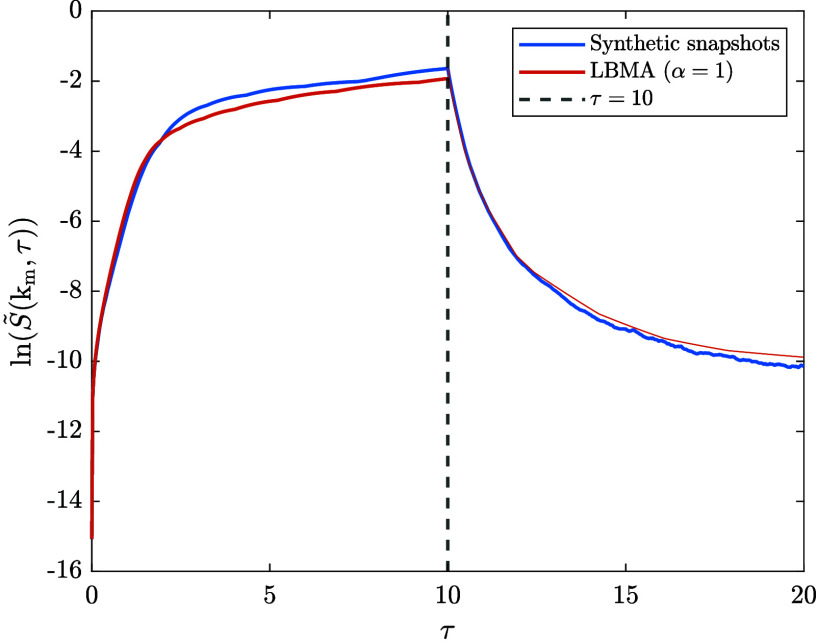
Illustration of the asymmetry of the time evolution of *S̃*(k_m_,τ) between spinodal decomposition (0 < τ ≤ 10) and dissolution (10 < τ ≤ 20) obtained by two methods: (1) using the synthetic structure factor snapshots from the dissolution time series, and (2) using the numerical solutions to the LBMA equation applied to spinodal decomposition and dissolution (with α = 1), respectively.

The asymmetry between the dynamics of spinodal decomposition and dissolution can be explained using the CHC-FHdG equation. In this equation, the amplification factor, which describes the rate of growth and decay of composition fluctuations, is given by

54 $$R\left(q\right)=\frac{-Mk_{B}T}{v_{0}}\left[2\left(\chi_{s}-\chi \right)q^{2}+\frac{\sigma^{2}}{18}\left(\frac{1}{\phi_{0}\left(1-\phi_{0}\right)}\right)q^{4}\right]$$

The first term is a diffusion-like term, which describes the transport of material driven by gradients in the chemical potential. The second term describes the effects of interfaces. In spinodal decomposition, χ > χ_*s*_. The diffusion and interface terms have different signs - they compete. In dissolution, χ < χ_*s*_. The diffusion and interface terms have the same sign - they combine. Putting this together, we see that, for a given |(χ_*s*_ – χ)|, the decay rate in dissolution is greater than the amplification rate in spinodal decomposition. Although this analysis neglects nonlinear effects, qualitatively we expect a similar asymmetry between spinodal decomposition and dissolution even in the mode-coupling regime. The LBMA equation predicts the following modified amplification factor:

55 R(q)[1+Z(q)]

As we have seen, the effect of the mode-coupling term, *Z*(*q*), is to enhance the decay rate in dissolution and slow the amplification rate in spinodal decomposition. Therefore, we expect that the qualitative conclusion from our analysis extends into the mode-coupling regime.

## Conclusions and Future Work

We tested the LBMA equation applied to dissolution and spinodal decomposition using synthetic structure factor snapshots and the linear CHC-FHdG equation.

For the most part, the LBMA equation applied to dissolution accurately described the dynamics of the synthetic structure factor snapshots, outperforming the linear CHC-FHdG equation. The increased accuracy of the LBMA equation over the linear CHC-FHdG equation can be traced back to the mode-coupling term in the former. We hope these findings motivate further testing of the LBMA equation applied to dissolution using experimental data. Contrasting our results, obtained using synthetic structure factor snapshots, with those of Akcasu et al.,^37^ obtained using experimental scattering data, we note that a prerequisite of any test using experimental data will be the ability to accurately determine the parameters contained in the equation. One advantage of using dissolution, rather than spinodal decomposition, to more thoroughly test the LBMA equation, is that different initial conditions can be generated by allowing phase separation to proceed for different time periods before taking the blend back into the one-phase region, without changing the temperature or composition. This allows for a more robust test of the LBMA equation since it will not be complicated by, possibly unknown, changes in the molecular and thermodynamic parameters required to model the process. In addition, this would enable the generation of sufficient data to take advantage of recent advances in the optimization of parameter estimation for integro-differential equations,^76^ such as the LBMA equation, which would overcome the challenge of uncertainty in the parameters used to model the time dependency of the structure factor.

While the LBMA equation applied to spinodal decomposition did not accurately describe the dynamics of the synthetic structure factor snapshots, it did capture some important qualitative features. For example, the decrease in the growth rate of the synthetic structure factor snapshots after the early stage and coarsening. The ability of the LBMA equation to capture these features can be traced back to the mode-coupling term. In contrast to the LBMA equation, the linear CHC-FHdG equation predicts the structure factor to grow exponentially and does not predict coarsening.

As shown recently by Sharratt et al.,^77^ experiments inside the one-phase region can be a powerful tool for testing linearized theories, such as the CHC-FHdG equation. Based on our results, we suggest that further exploration of the dynamics of dissolution will provide a stronger test of nonlinear theories, going beyond scaling laws, than has been possible with experiments probing spinodal decomposition. Experimental characterization of the asymmetry between the dynamics of spinodal decomposition and dissolution could also provide the foundation for the quantitative models that will be essential for guiding experimental development of materials derived from the manipulation of multistep temperature jumps,^78^ or from other methods of traversing phase diagrams^45,46^ to target desired morphologies. We hope our findings from testing the LBMA equation applied to spinodal decomposition and dissolution motivate further theoretical work aimed toward the development of a quantitative equation of motion for the structure factor during both processes.

## Supplementary Material



## Data Availability

The data from which the results in this paper can be derived are openly available in ORDA at 10.15131/shef.data.29307131.v1.

## Notes

Additional information about generating time series of synthetic structure factor snapshots (PDF)
