# Supplementary material for: Testing the Langer–Bar-on–Miller–Akcasu Equation for the Time Evolution of the Structure Factor during Polymeric Spinodal Decomposition and Dissolution
Source: Macromolecules. 2026 Apr 20;59(9):5203–18. doi: 10.1021/acs.macromol.6c00098 (PMC13173654; doi:10.1021/acs.macromol.6c00098)
Supplement: Supplementary file 1 [file ma6c00098_si_001.pdf]

Supporting information: testing the Langer - Bar-on - Miller - Akcasu equation for the time evolution of the structure factor during polymeric spinodal decomposition and dissolution

Matthew Jones[1,a)] and Nigel Clarke[1,b)]

[1]School of Mathematical and Physical Sciences, University of Sheffield, Hicks Building, Hounsfield Road, Sheffield, S3 7RH, United Kingdom
[a)]Now at Infinitesima Limited, Oxford, OX14 1RG, United Kingdom
[b)]n.clarke@sheffield.ac.uk

March 29, 2026

Introduction

This supporting information contains additional information about generating time series of synthetic structure factor snapshots. Equation, figure and table numbers with the prefix 'S' correspond to the supporting information. Those without correspond to the main paper.

S.1 Additional information about generating time series of synthetic structure factor snapshots

S.1.1 Deriving the key equations

Finite difference scheme (Eq. (43))

To begin the derivation, we nondimensionalise Eq. (4). We make use of the dimensionless variables in Eqs. (46a) - (46c). Upon substituting these dimensionless variables into Eq. (4), we obtain, after rearranging,

$$\frac{\partial \phi(\boldsymbol{x},\tau)}{\partial \tau} = \frac{1}{2}\tilde{\nabla}^2\Big[\frac{\chi_c}{2|\chi-\chi_s|}\ln\Big(\frac{\phi}{1-\phi}\Big) - \frac{2\chi\phi}{|\chi-\chi_s|} + \frac{1}{36}\Big(\frac{1-2\phi}{(\phi(1-\phi))^2}\Big)(\tilde{\nabla}\phi)^2 - 2\Big(\frac{1}{36\phi(1-\phi)}\Big)\tilde{\nabla}^2\phi\Big] + \tilde{\xi}(\boldsymbol{x},\tau). \quad \text{(S.1)}$$

Next, we write Eq. (S.1) in a slightly different form. A rescaled dimensionless noise term $\nu(\boldsymbol{x},\tau) = (\sigma^{3/2}/(v_0^{1/2}|\chi-\chi_s|^{1/4}))\tilde{\xi}(\boldsymbol{x},\tau)$ can be identified by substituting Eqs. (46a) - (46c) into Eq. (3b). Upon substituting $\tilde{\xi}(\boldsymbol{x},\tau) = (v_0^{1/2}|\chi-\chi_s|^{1/4}/\sigma^{3/2})\nu(\boldsymbol{x},\tau)$ into Eq. (S.1), we obtain

$$\frac{\partial \phi(\boldsymbol{x},\tau)}{\partial \tau} = \frac{1}{2}\tilde{\nabla}^2\Big[\frac{\chi_c}{2|\chi-\chi_s|}\ln\Big(\frac{\phi}{1-\phi}\Big) - \frac{2\chi\phi}{|\chi-\chi_s|} + \frac{1}{36}\Big(\frac{1-2\phi}{(\phi(1-\phi))^2}\Big)(\tilde{\nabla}\phi)^2 - 2\Big(\frac{1}{36\phi(1-\phi)}\Big)\tilde{\nabla}^2\phi\Big] + \frac{v_0^{1/2}|\chi-\chi_s|^{1/4}}{\sigma^{3/2}}\nu(\boldsymbol{x},\tau). \quad \text{(S.2)}$$

The first and second moments of $\nu(\boldsymbol{x},\tau)$ are given by

$$< \nu(\boldsymbol{x},\tau) >= 0 \quad \text{(S.3a)}$$

$$< \nu(\boldsymbol{x},\tau)\nu(\boldsymbol{x}',\tau') >= -\tilde{\nabla}^2\delta(\boldsymbol{x}-\boldsymbol{x}')\delta(\tau-\tau'). \quad \text{(S.3b)}$$

We note that a common simplification to Eq. (S.2) used in previous theoretical and computational studies of spinodal decomposition is to set v_0 equal to unity [1–4]. Similarly, in some studies, σ has also been set equal to unity [5–7].

Ultimately, we want to write down the numerical solution to Eq. (S.2). Therefore, following [2], we introduce some discretisation approximations. Specifically, we approximate continuous time as a series of discrete time steps of duration $\Delta\tau$ and continuous space as a simple cubic lattice (with periodic boundary conditions) comprising N_s^3 lattice sites of length Δx. We label the lattice sites with the indices (j,k,l) and the time steps with the index m. To approximate the spatial derivatives, we use central finite difference schemes. After integrating the resulting discretised equation over a single time step, we write the numerical solution as [2, 8–10]

$$\phi_{j,k,l}^{m+1} = \phi_{j,k,l}^{m} + \frac{\Delta\tau}{2\Delta x^2}\sum_{nn}\Big[\frac{\chi_c}{2|\chi-\chi_s|}\ln\Big(\frac{\phi_{j,k,l}^m}{1-\phi_{j,k,l}^m}\Big) - \frac{2\chi\phi_{j,k,l}^m}{|\chi-\chi_s|} + \frac{1}{36}\Big(\frac{1-2\phi_{j,k,l}^m}{(\phi_{j,k,l}^m(1-\phi_{j,k,l}^m))^2}\Big)\frac{1}{4\Delta x^2}\prod_{nn}\phi_{j,k,l}^m \quad \text{(S.4)}$$
$$- 2\Big(\frac{1}{36\phi_{j,k,l}^m(1-\phi_{j,k,l}^m)}\Big)\frac{1}{\Delta x^2}\sum_{nn}\phi_{j,k,l}^m\Big] + \frac{v_0^{1/2}|\chi-\chi_s|^{1/4}}{\sigma^{3/2}}M_{j,k,l}^m,$$

where $\sum_{nn}$ and $\prod_{nn}$ are the short-hand operators in Eqs. (44a) and (44b) and $M^m_{j,k,l} = \int_\tau^{\tau+\Delta\tau} \nu(\boldsymbol{x},\tau)d\tau$. We approximated the integral of the term with the square brackets as a Riemann sum with a single term.

To make use of Eq. (S.4), we must specify how to implement $M^m_{j,k,l}$. Following [9, 10], we use Eq. (S.3) to compute

$$< M^m_{j,k,l} >= 0 \tag{S.5a}$$

$$< M^m_{j,k,l} M^{m'}_{j',k',l'} >= -\tilde{\nabla}^2 \delta(\boldsymbol{x}-\boldsymbol{x}')\delta_{m,m'}\Delta\tau. \tag{S.5b}$$

Approximating the Laplacian using central finite differences and $\delta(\boldsymbol{x}-\boldsymbol{x}')$ as $\delta_{j,j'}\delta_{k,k'}\delta_{l,l'}/\Delta x^3$, we obtain

$$< M^m_{j,k,l} M^{m'}_{j',k',l'} >= -\frac{1}{\Delta x^2}\sum_{nn}\Big(\frac{\delta_{j,j'}\delta_{k,k'}\delta_{l,l'}}{\Delta x^3}\Big)\delta_{m,m'}\Delta\tau. \tag{S.6}$$

It can be shown that

$$< M^m_{j,k,l} M^{m'}_{j',k',l'} >= \begin{cases} \frac{6\Delta\tau}{\Delta x^5} & \text{if } (j,k,l)=(j',k',l') \text{ and } m=m' \\ \frac{-\Delta\tau}{\Delta x^5} & \text{if } (j,k,l) \text{ and } (j',k',l') \text{ are NN and } m=m' \\ 0 & \text{otherwise} \end{cases}, \tag{S.7}$$

where NN is an abbreviation for nearest neighbours. When $m = m'$, the second line on the right-hand side of this equation reveals the covariance of $M^m_{j,k,l}$ is negative for lattice sites that are nearest neighbours. Therefore, positive values of $M^m_{j,k,l}$ at one lattice site, which correspond to an increase in $\phi^m_{j,k,l}$, are compensated by negative values at the neighbouring lattice sites and vice versa. It follows that material is conserved. Ultimately, this conservation property stems from the presence of ∇^2 in Eq. (3b). Replacing ∇^2 in Eq. (3b) with a numerical factor results in the covariance term in the second line of Eq. (S.7) becoming zero. In this case, material would no longer be conserved.

To generate values of $M^m_{j,k,l}$ in accordance with Eqs. (S.5a) and (S.6), we follow [3, 9, 11] in defining

$$M^m_{j,k,l} = \frac{1}{\Delta x}\big[\eta^m_{1;j+1,k,l} - \eta^m_{1;j,k,l} + \eta^m_{2;j,k+1,l} - \eta^m_{2;j,k,l} + \eta^m_{3;j,k,l+1} - \eta^m_{3;j,k,l}\big], \tag{S.8}$$

where the η_n are independent Gaussian random variables with the following statistical properties:

$$< \eta^m_{n;j,k,l} >= 0 \tag{S.9a}$$

$$< \eta^m_{n;j,k,l}\eta^{m'}_{n';j',k',l'} >= \frac{\Delta\tau}{\Delta x^3}\delta_{n,n'}\delta_{j,j'}\delta_{k,k'}\delta_{l,l'}\delta_{m,m'}. \tag{S.9b}$$

Finally, upon substituting Eq. (S.8) into Eq. (S.4), we obtain Eq. (43).

Snapshots of the power spectrum and the structure factor (Eqs. (47) and (49))

To begin the derivation, we nondimensionalise Eq. (8). We make use of the dimensionless variables in Eqs. (46a), (50a) and (50b). Upon substituting these dimensionless variables into Eq. (8), we obtain, after rearranging,

$$\tilde{S}(\mathrm{k},\tau) = \frac{1}{\tilde{V}}\Big\langle \int d^3x\; e^{-i\mathbf{k}\cdot\boldsymbol{x}}\delta\phi(\boldsymbol{x},\tau)\int d^3x'\; e^{i\mathbf{k}\cdot\boldsymbol{x}'}\delta\phi(\boldsymbol{x}',\tau)\Big\rangle, \tag{S.10}$$

where $\tilde{V} = V|\chi-\chi_s|^{\frac{3}{2}}/\sigma^3$. We note that the expression inside the angle brackets is the dimensionless power spectrum $\tilde{P}(\mathrm{k},\tau)$, i.e. a dimensionless version of Eq. (9).

Next, consistent with the derivation of the finite difference scheme, we introduce discretisation approximations into Eq. (S.10). Most significantly, we approximate the Fourier transforms as discrete Fourier transforms. The resulting discretised equation can be expressed in two parts:

$$\tilde{S}_d^m = \frac{1}{N_s^3 \Delta x^3} < \tilde{P}_d^m > \tag{S.11a}$$

$$\tilde{P}_d^m = \Delta x^6 \Big\langle \sum_{j=0}^{N_s-1} \sum_{k=0}^{N_s-1} \sum_{l=0}^{N_s-1} \delta\phi_{j,k,l}^m \, e^{-\frac{2\pi i}{N_s}(aj+bk+cl)} \sum_{j'=0}^{N_s-1} \sum_{k'=0}^{N_s-1} \sum_{l'=0}^{N_s-1} \delta\phi_{j',k',l'}^{m'} e^{\frac{2\pi i}{N_s}(aj'+bk'+cl')} \Big\rangle_R, \tag{S.11b}$$

where a, b and c are integers in the range $-(N_s-1)/2 \leq a, b, c \leq (N_s-1)/2$, and $< ... >_R$ denotes a radial average. The radial average can be written explicitly as

$$< f_{a,b,c} >_R \equiv f_d = \frac{\sum_{a,b,c \text{ s.t. round}(\sqrt{a^2+b^2+c^2})=d} f_{a,b,c}}{\sum_{a,b,c \text{ s.t. round}(\sqrt{a^2+b^2+c^2})=d} 1}, \tag{S.12}$$

where d is an integer in the range $0 \leq d \leq (N_s-1)/2$. We introduce the radial average since $\tilde{P}(\mathrm{k}, \tau)$ depends only on the magnitude of $\mathbf{k}$ - we expect polymer blends to be isotropic during dissolution and spinodal decomposition. The integers a, b and c are related to $\mathbf{k}$ via $\mathbf{k} = (2\pi/(N_s\Delta x))(a, b, c)$. The integer d is related to $\mathrm{k} = |\mathbf{k}|$ via $\mathrm{k} = 2d\pi/(N_s\Delta x)$.

Next, we propose approximating $c \approx 0$ to make Eq. (S.11b) consistent with a small-angle scattering experiment. This corresponds to approximating $q_z \approx 0$, where q_z is the z-component of $\boldsymbol{q}$, and reduces the three-dimensional discrete Fourier transforms in Eq. (S.11b) to two-dimensional discrete Fourier transforms:

$$\tilde{P}_d^m = \Delta x^6 \Big\langle \sum_{j=0}^{N_s-1} \sum_{k=0}^{N_s-1} \sum_{l=0}^{N_s-1} \delta\phi_{j,k,l}^m \, e^{-\frac{2\pi i}{N_s}(aj+bk)} \sum_{j'=0}^{N_s-1} \sum_{k'=0}^{N_s-1} \sum_{l'=0}^{N_s-1} \delta\phi_{j',k',l'}^{m'} e^{\frac{2\pi i}{N_s}(aj'+bk')} \Big\rangle_R. \tag{S.13}$$

The radial average becomes

$$< f_{a,b} >_R \equiv f_d = \frac{\sum_{a,b \text{ s.t. round}(\sqrt{a^2+b^2})=d} f_{a,b}}{\sum_{a,b \text{ s.t. round}(\sqrt{a^2+b^2})=d} 1}. \tag{S.14}$$

Our logic behind approximating $q_z \approx 0$ is as follows. Defining the z-axis to be parallel to the incident beam, the scattering vector of an arbitrary scattering event is given by

$$\boldsymbol{q} = \boldsymbol{q}_f - \boldsymbol{q}_i = \begin{pmatrix} q_{f,x} \\ q_{f,y} \\ q_{f,z} \end{pmatrix} - \begin{pmatrix} 0 \\ 0 \\ q_{i,z} \end{pmatrix} = \begin{pmatrix} q_{f,x} \\ q_{f,y} \\ q_{f,z} - q_{i,z} \end{pmatrix}. \tag{S.15}$$

Assuming that the scattering is elastic (therefore $|\boldsymbol{q}_i| = |\boldsymbol{q}_f|$), it can be deduced using trigonometry that

$$q_z = |q_f|(\cos(\theta) - 1). \tag{S.16}$$

By definition, the scattering angle in small-angle scattering is small, therefore

$$q_z = |q_f|\Big(1 - \frac{\theta^2}{2!} + \frac{\theta^4}{4!} - ... - 1\Big) \approx 0. \tag{S.17}$$

Finally, for simplicity, we propose neglecting the time average represented by $< ... >$ in Eq. (S.11a). Instead, noting that the sizes of simulated blends are significantly smaller than

the sizes of blends studied experimentally, we propose taking $< ... >$ to mean an ensemble average [12,13]. This allows us to model the scattering throughout a 'large' experimental blend using N_r simulations of 'small' blends:

$$\tilde{S}_d^m = \frac{1}{N_r N_s^3 \Delta x^3} \sum_{n=1}^{N_r} \tilde{P}_{n;d}^m \tag{S.18a}$$

$$\tilde{P}_{n;d}^m = \Delta x^6 \Big\langle \sum_{j=0}^{N_s-1} \sum_{k=0}^{N_s-1} \sum_{l=0}^{N_s-1} \delta\phi_{n;j,k,l}^m \, e^{-\frac{2\pi i}{N_s}(aj+bk)} \sum_{j'=0}^{N_s-1} \sum_{k'=0}^{N_s-1} \sum_{l'=0}^{N_s-1} \delta\phi_{n;j',k',l'}^{m'} e^{\frac{2\pi i}{N_s}(aj'+bk')} \Big\rangle_R . \tag{S.18b}$$

These equations are equivalent to Eqs. (49) and (47), respectively.

S.1.2 Choosing suitable values of Δx and $\Delta\tau$ to use in the simulations

S.1.2.1 Background

When using finite difference simulations to generate data, one must be careful in the selection of Δx and $\Delta\tau$ values [2,11,14]. A guiding principle for choosing a suitable value of Δx is that it needs to be much less than the smallest physical length scale modelled in the system. The value of Δx places an upper limit on the value of $\Delta\tau$. In general, one can be confident they have used small enough values of Δx and $\Delta\tau$ when the data generated by a simulation is independent of these values, i.e. it does not change when smaller values are used.

S.1.2.2 Methodology

We used trial and error to choose the values of Δx and $\Delta\tau$ listed in the main paper. To facilitate this, we generated different versions of the spinodal decomposition and dissolution time series using several combinations of Δx, $\Delta\tau$, N_s and $m_{\max}$. For each 'type' of time series, i.e. spinodal decomposition or dissolution, we compared the time evolution of the synthetic structure factor snapshots corresponding to each combination of Δx, $\Delta\tau$, N_s and $m_{\max}$. We looked for consistent overlap between snapshots to determine which values of Δx and $\Delta\tau$ could be used to generate time series that are independent of these values. The specific combinations of Δx, $\Delta\tau$, N_s and $m_{\max}$ we investigated are listed in Table S.1. We chose the values of N_s and $m_{\max}$ to fix the size of the system and dimensionless timespan of the simulations, respectively.

Label	Δx	$\Delta\tau$	N_s	$m_{\max}$
A	1	1×10^{-3}	65	5×10^4
B	0.5	2.5×10^{-4}	129	2×10^5
C	0.25	6.25×10^{-5}	257	8×10^5
D	0.25	3.125×10^{-5}	257	1.6×10^6
E	0.2	4×10^{-5}	321	1.25×10^6

Table S.1: The combinations of discretisation parameters we used to search for suitable values of Δx and $\Delta\tau$ to use in the simulations. The labels are provided to make the table easier to refer to.

S.1.2.3 Results

First, we focus on the different versions of the dissolution time series. Figure S.1 compares the time evolution of the synthetic dissolution structure factor snapshots corresponding to each combination of Δx, $\Delta\tau$, N_s and $m_{\max}$ listed in Table S.1. For ease of reference, we use the labels in Table S.1 to distinguish between the different versions of the time series. The snapshots

corresponding to B, C, D and E overlap at each value of τ. For $\tau < 20$, there are discrepancies between these snapshots and the snapshots corresponding to A. These observations suggest that the versions of the dissolution time series corresponding to B, C, D and E are independent of the corresponding values of Δx and $\Delta\tau$, while the version corresponding to A is not.

We mention in passing that dissolution provides a straightforward opportunity to verify that we derived and coded Eqs. (43), (47) and (49) correctly. The synthetic structure factor snapshots should approach the curve given by Eq. (18), i.e. de Gennes' equation for the static structure factor, or, more specifically, a version of Eq. (18) that has been non-dimensionalised consistently with Eqs. (43), (47) and (49). In Fig. S.1f, the non-dimensionalised static structure factor is plotted alongside the synthetic structure factor snapshots. The static structure factor curve overlaps with the synthetic structure factor snapshots. This observation would be unlikely if we made a mistake in deriving or coding any of Eqs. (43), (47) and (49), therefore it indicates no mistakes were made. For $\tau > 15$, the synthetic structure factor snapshots exhibit fluctuations. We believe this effect stems from the Gaussian random variable term in Eq. (43).

We now focus on the different versions of the spinodal decomposition time series. Figure S.2 compares the time evolution of the synthetic spinodal decomposition structure factor snapshots corresponding to each combination of Δx, $\Delta\tau$, N_s and $m_{\max}$ listed in Table S.1. Again, we use the labels in Table S.1 to distinguish between the different versions of the time series. The snapshots corresponding to C, D and E overlap at each value of τ. For $\tau < 5$, there are discrepancies between these snapshots and the snapshots corresponding to B, and, for all values of τ, there are discrepancies between these snapshots and the snapshots corresponding to A. These observations suggest that the versions of the spinodal decomposition time series corresponding to C, D and E are independent of the corresponding values of Δx and $\Delta\tau$, while the versions corresponding to A and B are not.

To conclude, we found the following combinations of Δx and $\Delta\tau$ values can be used to generate versions of the dissolution and spinodal decomposition time series that are independent of these values: $\Delta x = 0.25$ & $\Delta\tau = 6.25 \times 10^{-5}$ (C), $\Delta x = 0.25$ & $\Delta\tau = 3.125 \times 10^{-5}$ (D) and $\Delta x = 0.2$ & $\Delta\tau = 4 \times 10^{-5}$ (E).

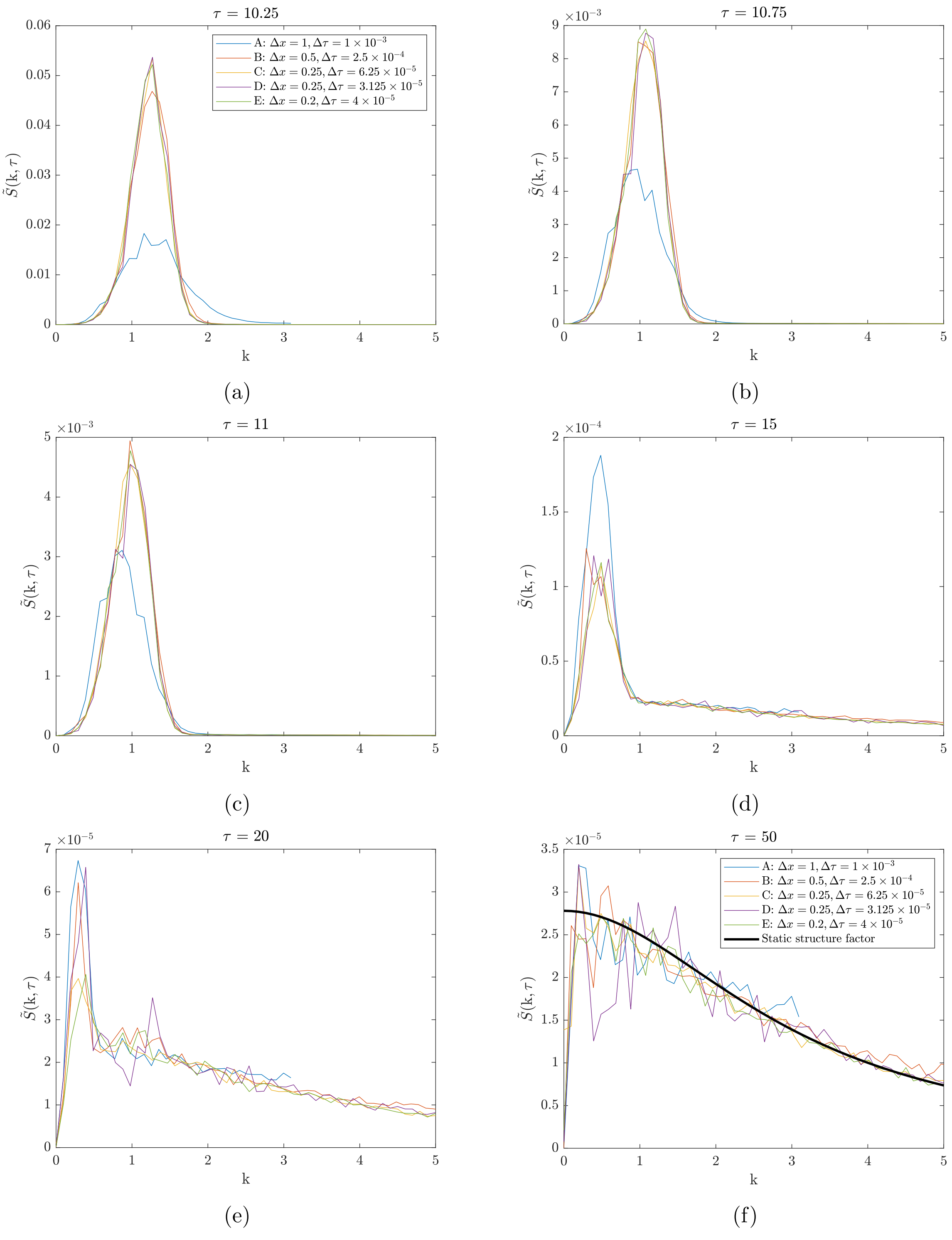

Figure S.1: A comparison between snapshots of the synthetic structure factor generated using different combinations of Δx, $\Delta\tau$, N_s and $m_{\max}$ in the simulations of dissolution. In panel (f), a non-dimensionalised version of de Gennes' equation for the static structure factor (Eq. (18)) is plotted. In the simulations of dissolution, dissolution was initiated at $\tau = 10$. The data in the figure corresponds to times that lag behind the onset of dissolution by 0.25, 2, 4, 8, 16 and 32 dimensionless time units, respectively.

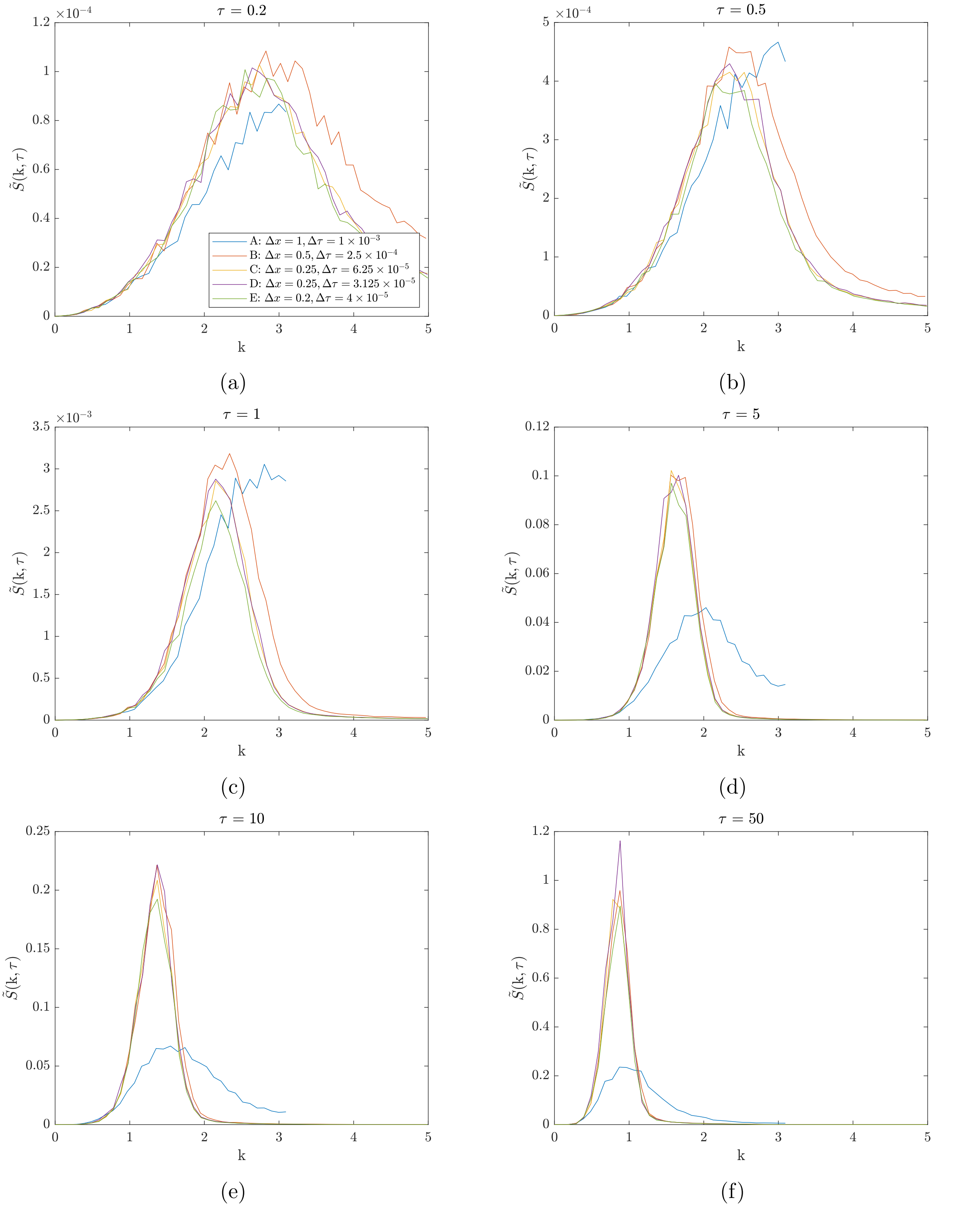

Figure S.2: A comparison between snapshots of the synthetic structure factor generated using different combinations of Δx, $\Delta\tau$, N_s and $m_{\max}$ in the simulations of spinodal decomposition.

S.1.3 Quantifying the the small-k limit of Eq. (43)

The small-k limit of Eq. (43) stems from deriving the coefficient to $|\nabla\phi|^2$ in Eq. (2) (known as the 'square gradient coefficient') to be consistent with the small-q limit of de Gennes' random phase approximation. Specifically, instead of using complete Debye functions in the derivation of the square gradient coefficient, de Gennes used its linear expansion, which is only valid in the limit $qR_g = \mathrm{k}r_g << 1$ [15–18]. We attempt to quantify this limit below.

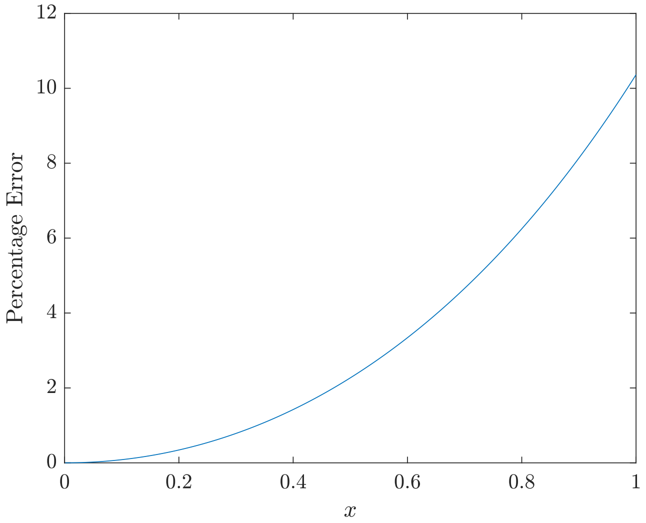

Figure S.3: The percentage error between the full Debye function and its linear expansion as a function of $x = (qR_g)^2 = (\mathrm{k}r_g)^2$.

The dimensionless radius of gyration is given by

$$r_g = \sqrt{\frac{N|\chi - \chi_s|}{6}}. \tag{S.19}$$

Therefore, the limit $\mathrm{k}r_g << 1$ can be written as

$$\mathrm{k}\sqrt{\frac{N|\chi - \chi_s|}{6}} << 1. \tag{S.20}$$

Substituting $N = 2700$ and the values of χ and χ_s corresponding to each time series into Eq. (S.19), we determine that

- $r_g \approx 0.10 \implies \mathrm{k} << 10$ for both the spinodal decomposition and dissolution time series.

In an attempt to quantify what is meant by '<<', we turn to the Debye function and its linear expansion:

$$f_D(x) = \frac{2}{x^2}\left(x - 1 + e^{-x}\right) \approx 1 - \frac{x}{3}, \tag{S.21}$$

where $x = (qR_g)^2 = (\mathrm{k}r_g)^2$. Figure S.3 shows the percentage error between the Debye function and its linear expansion as a function of x. At $x = 0.5$, the percentage error is approximately 2.22 %, which suggests the linear expansion is a good approximation to the full Debye function when $x \leq 0.5$. Using $x \leq 0.5$ and the values of r_g listed above, we determine the following small-k limit inequalities:

- $\mathrm{k} < 7$ for both the spinodal decomposition and dissolution time series.

References

[1] K. Binder. Collective diffusion, nucleation, and spinodal decomposition in polymer mixtures. *The Journal of Chemical Physics*, 79(12):6387–6409, 1983.

[2] S. C. Glotzer. Computer Simulations of Spinodal Decomposition in Polymer Blends. In *Annual Reviews of Computational Physics II*, pages 1–46. World Scientific, 1995.

[3] A. Aksimentiev, K. Moorthi, and R. Holyst. Scaling properties of the morphological measures at the early and intermediate stages of the spinodal decomposition in homopolymer blends. *Journal of Chemical Physics*, 112(13):6049–6062, 2000.

[4] M. Fiałkowski and R. Hołyst. The unphysical pinning of the domain growth during the separation of homopolymer blends near the spinodal. *The Journal of Chemical Physics*, 120(12):5802–5808, 2004.

[5] A. Chakrabarti, R. Toral, J. D. Gunton, and M. Muthukumar. Spinodal Decomposition in Polymer Mixtures. *Physical Review Letters*, 63(19):2072–2075, 1989.

[6] A. Chakrabarti, R. Toral, J. D. Gunton, and M. Muthukumar. Dynamics of phase separation in a binary polymer blend of critical composition. *The Journal of Chemical Physics*, 92(11):6899–6909, 1990.

[7] J. D. Gunton, R. Toral, and A. Chakrabarti. Numerical Studies of Phase Separation in Models of Binary Alloys and Polymer Blends. *Physica Scripta*, T33:12–19, 1990.

[8] L. Ramírez-Piscina, J. M. Sancho, and A. Hernández-Machado. Numerical algorithm for Ginzburg-Landau equations with multiplicative noise: Application to domain growth. *Physical Review B*, 48(1):125, 1993.

[9] R. Petschek and H. Metiu. A computer simulation of the time-dependent Ginzburg–Landau model for spinodal decomposition. *The Journal of Chemical Physics*, 79(7):3443–3456, 1983.

[10] R. Toral and P. Colet. *Stochastic Numerical Methods: An Introduction for Students and Scientists.* Wiley-VCH, 2014.

[11] T. M. Rogers, K. R. Elder, and R. C. Desai. Numerical study of the late stages of spinodal decomposition. *Physical Review B*, 37(16):9638–9649, 1988.

[12] R. Roe. *Methods of X-ray and neutron scattering in polymer science.* Oxford University Press, 2000.

[13] J. S. Higgins and H. C. Benoit. *Polymers and Neutron Scattering.* Oxford University Press, 1994.

[14] C. Castellano and S. C. Glotzer. On the mechanism of pinning in phase-separating polymer blends. *J. Chem. Phys*, 103(21):9363–9369, 1995.

[15] G. R. Strobl. Structure Evolution during Spinodal Decomposition of Polymer Blends. *Macromolecules*, 18(3):558–563, 1985.

[16] P. G. de Gennes. *Scaling Concepts in Polymer Physics.* Cornell University Press, 1979.

[17] A. Z. Akcasu and I. C. Sanchez. A closed-form, free-energy functional for a binary polymer mixture. *Journal of Chemical Physics*, 88(12):7847–7850, 1988.

[18] R. A. L. Jones and R. W. Richards. *Polymers at Surfaces and Interfaces.* Cambridge University Press, 1999.
